# Quantitative comparative study on the verb-direction constructions “V+ *Xia* (下)”, “V+ *Xialai* (下来)” and “V+ *Xiaqu* (下去)” in mandarin Chinese

**DOI:** 10.1371/journal.pone.0354793

**Published:** 2026-07-29

**Authors:** Tian Tan, Jiangcai Li

**Affiliations:** 1 College of International Education, Shandong University, Jinan, Shandong, China; 2 College of Liberal Arts, Changsha, Hunan, China; SRMIST (Deemed to be University): SRM Institute of Science and Technology (Deemed to be University), INDIA

## Abstract

“V+ *Xia* (下)”, “V+ *Xialai* (下来)” and “V+ *Xiaqu* (下去)” are common verb-direction constructions in Mandarin Chinese, exhibiting certain similarities in syntactic distribution and semantic content. Employing collostructional analysis, this study quantitatively and visually calculates the collocational strength and attraction between different types of verbs and these verb-direction constructions. It analyzes the polysemy of the three constructions, summarizes the differences in their prototype meaning, and examines the distribution of collocational strength and relative semantic distance between prototypical and non-prototype meanings. Furthermore, it explores the characteristics of the semantic network structures presented by the three constructions. The findings indicate that “V+ *Xialai*” possesses the largest number of attracted verbs, the broadest verb selection range, and the highest productivity among the three constructions. “V+ *Xia*”, constrained by prosody, exhibits the lowest productivity but the highest average collocational strength. “V+ *Xia*” has the highest number of semantic types with a relatively uniform distribution of collocational strength, whereas the distributions for “V+ *Xialai*” and “V+ *Xiaqu*” are more concentrated around their prototype meanings. The semantic networks of “V+ *Xia*” and “V+ *Xiaqu*” overall display a gradient continuum, while that of “V+ *Xialai*” is relatively discrete, with certain semantic types showing a tendency to form independent clusters. The prototype meanings of all three constructions demonstrate strong semantic coherence. Non-prototype meanings with high collocational strength also exhibit relatively strong internal coherence, while those with low collocational strength are more discrete, displaying characteristics of diversity.

## Introduction

In Modern Mandarin, *Xia* can function independently as a directional verb, or it can combine with *Lai* and *Qu* to form the complex directional verbs *Xialai* and *Xiaqu*. All three elements can collocate with a verb to yield the verb-direction constructions “V+ *Xia*”, “V+ *Xialai*” and “V+ *Xiaqu*”. In these constructions, the label “verb” refers to a “verb schema” or, more generally, a verbal element; in terms of word class, it is not restricted to verbs proper—under appropriate contextual conditions, certain nouns or adjectives that possess verbal properties may also serve as the verbal element (e.g., *Hongshang* (红上), *Huoshang* (火上), etc.) [1]. Syntactically, the three verb-direction constructions draw on largely overlapping sets of verbs, and in some contexts they can be substituted for one another without loss of grammaticality. Semantically, all three can express displacement from a higher to a lower position. For example:

**Table:** 

(1)	a.	帷幕	缓缓	落下
		wéi mù	huǎn huǎn	luò xià
		curtain	slowly	go down
	b.	帷幕	缓缓	落下来
		wéi mù	huǎn huǎn	luò xià lái
	c.	帷幕	缓缓	落下去
		wéi mù	huǎn huǎn	luò xià qù
		The curtain came down slowly.		

Regarding the verb-direction constructions “V+ *Xia*”, “V+ *Xialai*” and “V+ *Xiaqu*”, current academic discussions in the field mainly focus on the following five aspects: the contrast between *Shang* and *Xia*, as well as the research on symmetry and asymmetry between *Shang*-type verb-direction constructions and *Xia*-type verb-direction constructions [2–4]; the various semantic types of verb-direction constructions and the connections between their specific meanings [5–7]; the sentence-embedding patterns and pragmatic functions of verb-direction constructions [8–10]; the investigation into the formation and diachronic evolution of verb-direction constructions [11–13]; and the acquisition and error analysis of verb-direction constructions [14–15].

The abundant existing research findings have systematically revealed the syntactic and semantic features, diachronic evolution paths, and acquisition rules of the verb-direction constructions “V+ *Xia*”, “V+ *Xialai*” and “V+ *Xiaqu*”, which hold significant theoretical value for subsequent studies. However, the core conclusions of existing research mainly rely on researchers’ “intuition” and “introspection”, lacking empirical support from a large amount of linguistic data. Methodologically, it is difficult to explain linguistic phenomena in a comprehensive and objective manner. In recent years, scholars in Chinese linguistics have increasingly recognized this limitation and have begun to strengthen the empirical foundation of their research with corpus data. Yang and Li, for example, conduct a diachronic corpus-based study of the directional complement *Guolai*(过来), drawing on data spanning 15 historical periods from the CCL Corpus and examining its semantic evolution from the macro-event perspective [16]. The present study follows this empirical turn but shifts the focus to synchronic analysis, applying the collostructional analysis approach to address the following three questions in a quantitative and visualization-based manner:

1. What are the collocational strength values between the verbs selectable for the verb-direction constructions “V+ *Xia*”, “V+ *Xialai*” and “V+ *Xiaqu*” and the constructions themselves? How many verbs exhibit an attraction relationship with the constructions? And what collocational tendencies do the verbs show across the constructions?
2. What are the prototype meanings of the verb-direction constructions “V+ *Xia*”, “V+ *Xialai*” and “V+ *Xiaqu*”? What is the proportion of collocational strength for each semantic type? And what is the relative semantic distance between the prototype meanings and non-prototype meanings?
3. What kind of constructional semantic networks do the verb-direction constructions “V+ *Xia*”, “V+ *Xialai*” and “V+ *Xiaqu*” present? And what commonalities and differences do they exhibit in terms of the distribution of constructional meanings?

## Theoretical framework

### Usage-based constructionist view of language and its methodology

Langacker was the first to propose the “usage-based model,” arguing that linguistic knowledge originates from and is rooted in language use, and emphasizing that language acquisition and use are experience-driven [17]. Building on this foundation, Goldberg further put forward the “usage-based constructionist approach” [18]. She holds that the basic unit of language is the construction, and a speaker’s linguistic competence is reflected in the vast, hierarchically rich construction network stored in their mental grammar. The frequency of language use plays a core role in the formation, representation, and change of linguistic knowledge.

Goldberg defines a construction as a form-meaning pairing that cannot be strictly predicted from its component parts or from previously existing constructions. Such pairings can range in scale from a single morpheme to a syntactic structure. The research objects of this paper—the verb-direction constructions “V+ *Xia*”, “V+ *Xialai*”, and “V+ *Xiaqu*”—are traditional form-meaning pairs: their meanings cannot be strictly predicted from their components, and they have a high frequency of use in modern Chinese. Therefore, they can be regarded as constructions.

The usage-based constructionist view of language advocates a set of methodologies that are highly consistent with its theoretical principles, with the core lying in the empirical investigation of language use. In contemporary linguistics, the analysis of actual language use mainly relies on corpus data, which is then presented with the help of quantitative and statistical methods. Hilpert surveys the current landscape of Construction Grammar and identifies the integration of corpus-based quantitative methods, network models of constructional organization, and the central role of frequency as the most promising avenues for future research [19]. Gries argues that there is a symbiotic relationship between usage-based linguistics and corpus methodology. Corpus methodology is empirical: its research conclusions are obtained through probabilistic statistical procedures based on a large number of authentic texts, and it can address questions that are difficult to explain using experiential methods [20].

### Family resemblances and prototype theory

“Family resemblances” and “prototype” are two extremely important and interrelated concepts in cognitive linguistics. The concept of “family resemblances” was first proposed by Wittgenstein [21]. Its core idea is that members of a category do not share a set of common features that all members possess; instead, members are connected through a network of overlapping and intersecting similarity features—much like the way members of a family are connected through shared facial characteristics. Furthermore, the boundaries of a category are vague and open, with no clear demarcation.

Prototype theory was first put forward by Rosch [22]. She argues that a category is organized around a typical example (the prototype) or a set of typical features (prototype features). The prototype is the most representative and typical member of the category. It does not necessarily exist in reality and can be a mental representation. Members within a category have unequal status: non-prototype members possess different degrees of membership based on their similarity to the prototype.

Goldberg holds that constructions are polysemous families with interconnected meanings, and that prototype theory can be fruitfully applied to their analysis. Within a construction’s multiple semantic types, one typically serves as the prototype. Prototypes emerge naturally from repeated linguistic instances, and in usage‑based theory, a form’s frequency of use is taken as a direct indicator of its degree of cognitive entrenchment [23]. Therefore, counting and quantifying usage frequency is of great significance for understanding constructions [24].

### Collostructional analysis

Key concepts related to the frequency of language construction use mainly include token frequency and type frequency. Token frequency refers to the total number of actual occurrences of a specific linguistic form (which can be a lexical item, phrase, or construction instance) in a corpus or language use. Token frequency is usually more closely associated with specific, concrete linguistic instances; forms with high token frequency are more easily retrieved automatically in mental representation and are more resistant to analogical change or regularization. Type frequency refers to the number of different lexical items (types) that can fill a specific slot in a particular construction. It measures the lexical diversity or productive potential of that slot.

Collostructional analysis is a set of corpus linguistics methods developed by Stefanowitsch and Gries by integrating cognitive linguistics theories [25]. Its main function is to quantify and test the collocational strength between specific lexical items and specific constructions, as well as the interactive relationship of attraction or repulsion between them. This method effectively avoids the errors of traditional statistical methods (such as Z-score, T-score, chi-square test, etc.)—errors that easily arise because these traditional methods only focus on the co-occurrence between words and are thus affected by natural language data failing to meet the requirements of normal distribution and homogeneity of variance. Collostructional analysis adopts Fisher’s exact test, which determines collocational strength by observing whether a target item appears more frequently in a specific construction than expected, without distributional assumptions. It then judges whether the relationship between the item and the construction is one of attraction or repulsion, and is therefore more accurate. The empirical validity of this method has been confirmed across diverse linguistic phenomena. For instance, Newman demonstrates that collostructional analysis reliably uncovers collocational preferences that traditional frequency-based measures often miss [26]. Its applicability to Chinese has been further validated by Liao, Gries and Wulff, who successfully employ the quantitative tools of collostructional analysis to capture subtle semantic distinctions among competing dative constructions in Mandarin Chinese [27].

Collostructional analysis includes three quantitative tools: collexeme analysis, distinctive collexeme analysis, and covarying collexeme analysis. The research objects of this paper—“V+ *Xia*”, “V+ *Xialai*” and “V+ *Xiaqu*”—have similar meanings and each contains only one slot; therefore, this study mainly employs the first two tools: Collexeme analysis is used to identify which lexical items appear significantly more frequently than (attraction) or significantly less frequently than (repulsion) their overall expected frequency in the corpus; Distinctive collexeme analysis is used to compare constructions that are similar in meaning or function, and to identify which lexical items show a significant preference for appearing in one of these constructions.

## Materials and methods

### Corpus source

This study selects the BCC Corpus of Beijing Language and Culture University as its corpus source, and observes authentic language usage examples in the corpus for frequency statistics. The BCC Corpus is a large-scale online database dominated by Chinese, serving the research on language ontology and language application. With a total scale of approximately 9.5 billion characters, it supports wildcard searches with part-of-speech(POS) tags and split-morpheme pattern searches, and can provide statistical data and example support for linguistic research [28].

### Calculation method

The BCC Corpus supports searches with POS information. For the research objects of this paper, searches can be conducted using the queries “v*Xia* (v下)”, “v*Xialai* (v下来)” and “v*Xiaqu* (v下去)”, and the “Statistics” section of the search results will display all lexical items that can fill the “V” slot.

Non‑directional homographs were disambiguated through a three‑stage procedure. First, all search queries required the slot‑filling element to be verb‑tagged, automatically excluding noun‑*Xia* and adjective‑*Xia* sequences. Second, two types of non‑directional verb + *Xia* sequences were excluded using the “Filter” function in the search results interface. (i) The “*Zai* (在)... *Xia*” construction, where *Xia* functions as a locative particle, was excluded by entering the queries “*Zai*nv*Xia* (在nv下)” and “*Zai*n*De*(的)v*Xia* (在n的v下)” in the filter bar. (ii) Cases where *Xia* is a shortened form of *Yixia* (一下) serving as a modal particle were excluded by entering common collocations such as “*Peihe*(配合)*Xia*”, “*Kaolv* (考虑)*Xia*”, “*Jieshi* (解释)*Xia*”, “*Anwei* (安慰)*Xia*”, “*Fangsong* (放松)*Xia*”, “*Qingzhu* (庆祝)*Xia*”, and “*Kaixin* (开心)*Xia*” in the filter bar. Third, the few remaining ambiguous cases were resolved through manual screening.

The basic form of a verb-direction construction is “verb + directional verb”. In addition to this basic form, verb-direction constructions also include potential forms and split forms. Potential forms are those with *De* (得) or *Bu* (不) inserted between the verb and the directional verb, and were retrieved using the queries “v*DeXiaLai*”(v得下来), “v*BuXiaLai*”(v不下来), “v*DeXiaQu*”(v得下去), and “v*BuXiaQu*”(v不下去). Split forms are those with a locative or patient argument inserted either within the directional verb or between the verb and the directional verb, and were retrieved using the queries “v*Xia*n*Lai*”(v下n来), “v*Xia*n*Qu*”(v下n去), “vn*XiaLai*”(vn下来), and “vn*XiaQu*”(vn下去). After removing garbled text and duplicate data, qualified verb-direction constructions were further screened through manual inspection and annotated for their semantic types.

The manual screening and subsequent semantic type annotation of verb-direction constructions were carried out by two annotators with backgrounds in linguistics. Prior to the formal annotation, both annotators received training on the criteria for identifying verb‑direction constructions, including the scope of the category and common ambiguous cases. A pilot annotation was first conducted on a sample of 100 instances. Annotation disagreements were then resolved through adjudication, and inter-annotator consistency was assessed using Cohen’s kappa (κ = 0.87 for verb-direction construction identification; κ = 0.79 for semantic type annotation). Formal annotation proceeded after acceptable agreement was achieved.

Below are two representative examples of ambiguous cases encountered during annotation, illustrating the necessity of contextual disambiguation. The first type concerns the “V+ *Xia*” form, where *Xia* may not function as a directional verb but can instead serve as a verbal measure word, indicating a brief or tentative sense (equivalent to ‘a bit’ or ‘once’). For example:

**Table:** 

(2)	a.	那	件	衣服	挂	得	很	高	，
		nà	jiàn	yī fu	guà	de	hěn	gāo	
		that	piece	clothing	hung	DER	very	high	
		你	帮	我	取	下	它	吧	。
		nǐ	bāng	wǒ	qǔ	xià	tā	ba	
		2SG	help	1SG	take	down	3SG	SP	
		That piece of clothing is hung too high, could you help me take it down.							
	b.	我	的	快递	送	到	代收点	了	，
		wǒ	de	kuài dì	sòng	dào	dài shōu diǎn	le	
		1SG	‘s	express delivery	deliver	to	pickup point	PFV	
		你	帮	我	取	下	它	吧	。
		nǐ	bāng	wǒ	qǔ	xià	tā	ba	
		2SG	help	1SG	take	one time	3SG	SP	
		My express delivery has been delivered to the pickup point, could you please help me pick it up.							

In both (2a) and (2b), the string *Quxia*(取下) is identical in form but differs in meaning. When the preceding context provides height-related information (“hung too high” as in (2a)), *Xia* in *Quxia* acts as a directional complement, denoting downward movement of the object; in such cases, *Quxia* is classified as a verb-direction construction. By contrast, when the context merely involves taking or fetching an item that is not positioned at a height, as in (2b), where the parcel is simply collected from a pickup point, *Xia* is interpreted as a verbal measure word and the sequence is not treated as a verb-direction construction. Hence, determining whether this type of “V+ *Xia*” constitutes a verb-direction construction requires taking contextual information into account.

The second type of ambiguity involves the “V+ *Xiaqu*” form. A linear “V+ *Xiaqu*” sequence does not always instantiate a verb-direction construction of the “V+ *Xiaqu*” type; it can instead be composed of a “V+ *Xia*” verb-direction construction followed by the purposive verb *Qu* in a serial verb construction. Consider example (3):

**Table:** 

(3)	把	零件	拆	下	去	洗	干净	。
	bǎ	líng jiàn	chāi	xià	qù	xǐ	gān jìng	
	P	parts	disassemble	apart	to	wash	clean	
	Disassemble the parts and clean them thoroughly.							

In (3), *Chaixiaqu*(拆下去) is not a unitary “V+ *Xiaqu*” construction. The internal structure is analyzed as follows: *Chaixia* is a “V+ *Xia*” verb-direction construction serving as the predicate, meaning “disassemble (the parts)”, while *Qu* functions as a purposive verb in a serial verb construction, introducing the subsequent action *Xiganjing* (洗干净, “wash clean”). The entire sentence thus means “Disassemble the parts and then wash them clean”. Consequently, when annotators encounter a “V+ *Xiaqu*” sequence, they need to determine whether *Qu* is an independent serial verb marker in order to avoid erroneously labeling the string as a single verb-direction construction.

After the manual inspection, we performed annotations and statistics according to the data categories required by the collostructional analysis method. Subsequently, the collostructional analysis script Coll. analysis 4.1 developed by Gries is used to perform calculations in the R Environment.

### Collexeme analysis

Collexeme analysis measures the degree of association between a target word and a construction, which requires counting five types of frequencies: the frequency of the target word in the target construction (a); the frequency of non-target words in the target construction (b); the frequency of the target word in non-target constructions (c); the frequency of non-target words in non-target constructions (d); and the total frequency of the corpus (W). See Table 1.

**Table 1:** Data Categories to Be Counted in Collexeme Analysis.

	Target Construction	Non-Target Constructions	Total
**Target Word**	a	c	a + c
**Non-Target Words**	b	d	b + d
**Total**	a + b	c + d	W = a + b + c + d

Using the five types of frequencies in Table 1, the significance of the observed frequency a is calculated through the hypergeometric distribution via the Fisher’s exact test formula:

$\begin{array}{c} p=\frac{(\text{a}+\text{b})!\cdot (\text{c}+\text{d})!\cdot (\text{a}+\text{c})!\cdot (\text{b}+\text{d})!}{\text{W}!\cdot \text{a}!\cdot \text{b}!\cdot \text{c}!\cdot \text{d}!} \end{array}$

In Fisher’s exact test, 0.05 is generally used as the threshold for the significance level. That is, when p < 0.05, there is a significant correlation between the verb and the construction; moreover, the smaller the p-value, the higher the degree of association. To account for multiple comparisons, the Benjamini‑Hochberg false discovery rate (FDR) correction was applied to the resulting p‑values. All reported significant attractions are based on the adjusted p‑values.

### Distinctive collexeme analysis

Distinctive collexeme analysis can reveal subtle differences in lexical selection preferences between two competing constructions. When comparing three groups of constructions, it is necessary to conduct tests in pairs, and four types of frequencies need to be counted: the frequency of the target word in Construction 1 (a); the frequency of the target word in Construction 2 (b); the total frequency of all words in Construction 1 (N₁); and the total frequency of all words in Construction 2 (N₂). See Table 2.

**Table 2:** Data Categories to Be Counted in Distinctive Collexeme Analysis.

	Construction 1	Construction 2	Total
**Target Word**	a	b	a + b
**All Other Words**	N₁-a	N₂-b	(N₁ + N₂)-(a + b)
**Total**	N₁	N₂	N₁ + N₂

Using the four types of frequencies in Table 2, the Fisher’s exact test formula is applied to calculate whether the distribution of the target word in Construction 1 and Construction 2 significantly deviates from random expectations:

p=∑k=amin(a+b,N1)(a+bk)((N1+N2)−(a+b)N1−k)(N1+N2N1)

For the test result, the p-value also uses 0.05 as the threshold for the significance level. When p < 0.05, the collocational tendency of the verb across the constructions is significant.

## Results and discussion

### Analysis of collocational strength between selected verbs and the constructions “V+ *Xia*”, “V+ *Xialai*” and “V+ *Xiaqu*”

When calculating the degree of association between words and constructions in the R language environment, some collocations may have excessively small test p-values, making them inconvenient to read. Gries performs a negative logarithmic transformation on p-values with base 10, i.e., -lg(p), which is recorded as “collocational strength” [29]. The smaller the p-value, the greater the collocational strength and the higher the degree of association between the word and the construction. Since 0.05 is the threshold for significance, -lg(0.05) ≈ 1.301; therefore, when the collocational strength is greater than 1.301, there is a significant attraction relationship between the verb and the construction.

**Fig 1:**
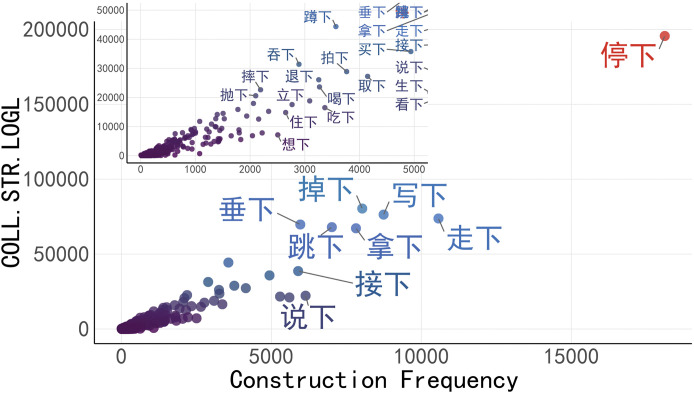
The distribution of collocational strength of optional verbs in the construction “V+ *Xia*”.

Through collexeme analysis of the collected relevant frequencies, it can be found that: the verb-direction construction “V+ *Xia*” has a total of 855,667 corpus entries in the corpus, with 621 types of words that can fill the verb slot of the construction. Among these, 396 verbs show a significant attraction relationship with “V+ *Xia*”. The corresponding relationship between verb frequency and collocational strength is detailed in Fig 1.

Due to space constraints, only the top 20 verbs with the highest collocational strength with “V+ *Xia*” and their specific strength values are listed here. As shown in Table 3 (full collocational strength results for all attracted verbs are provided in Appendix 1).

**Table 3:** Data list of 20 collocations with the strongest relevance to “V+ Xia” construction.

Rank	Word	Frequency	collocational strength	Rank	Word	Frequency	collocational strength
1	*Ting* (停)	18116	195602.9	11	*Tun* (吞)	2893	31403.4
2	*Diao* (掉)	8030	80412.8	12	*Pai* (拍)	3765	28882.2
3	*Xie* (写)	8743	76367.6	13	*Qu* (取)	4148	27247.8
4	*Zou* (走)	10571	73783.1	14	*Tui* (退)	3255	26083.0
5	*Chui* (垂)	5965	69752.1	15	*He* (喝)	3269	23613.3
*6*	*Tiao* (跳)	7017	67993.0	16	*Shuai*（摔)	2191	22635.5
7	*Na*（拿)	7822	67228.5	17	*Sheng*（生)	5290	21651.2
8	*Dun*（蹲）	3568	44353.8	18	*Pao*（抛）	2102	20586.4
9	*Jie*（接）	5894	38672.0	19	*Li*（立）	3089	18818.1
10	*Mai*（买）	4939	35783.3	20	*Hua*（滑）	2061	17950.6

It can be observed from Fig 1 and Table 3 that the collocational strength between verbs and the construction is not completely positively correlated with verb frequency. The frequencies of *“Dun Xia”* and *“Tun Xia”* collected in the corpus are lower than those of *“Qu Xia”* and *“Sheng Xia”*. However, collexeme analysis takes into account the overall frequency of collocational words in the corpus. After calculation, it is found that since the overall usage frequencies of *Dun* and *Tun* are much lower than those of *Qu* and *Sheng*, their collocational strength with the “V+ *Xia*” construction is higher.

**Fig 2:**
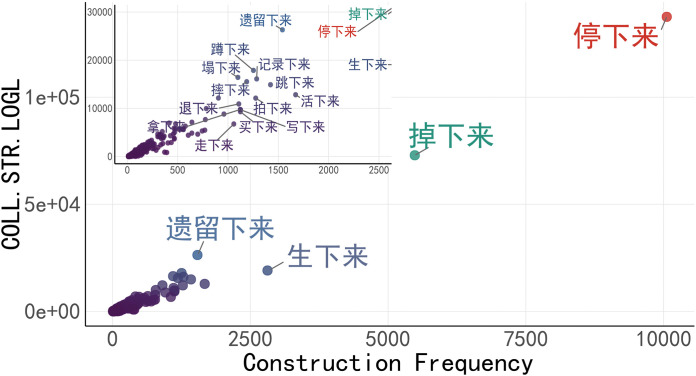
The distribution of collocational strength of optional verbs in the construction “V+ *Xialai*”.

**Fig 3:**
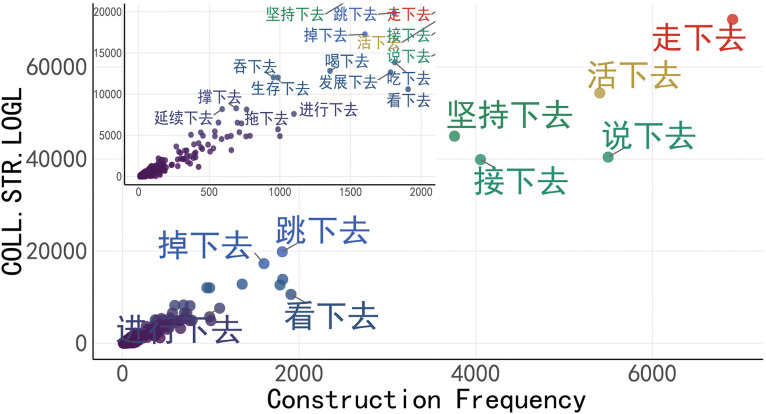
The distribution of collocational strength of optional verbs in the construction “V+ *Xiaqu*”.

By conducting statistics and calculations on “V+ *Xialai*” and “V+ *Xiaqu*” in the same way, the following results are obtained: “V+ *Xialai*” has a total of 114,975 corpus entries in the corpus. There are 770 types of words that can fill the verb slot of the construction, among which 554 verbs show a significant attraction relationship with “V+ *Xialai*”. “V+ *Xiaqu*” has a total of 114,745 corpus entries in the corpus. There are 711 types of words that can fill the verb slot of the construction, among which 495 verbs show a significant attraction relationship with “V+ *Xiaqu*”. The corresponding distribution of verb frequency and collocational strength, as well as the specific strength values of the top 20 collocational words with the highest correlation with the two constructions, are listed in Figs 2, 3 and Tables 4,5 (full collocational strength results for all attracted verbs are provided in Appendix 2–3).

**Table 4:** Data list of 20 collocations with the strongest relevance to “V+ Xialai” construction.

Rank	Word	Frequency	collocational strength	Rank	Word	Frequency	collocational strength
1	*Ting*(停)	10057	137543.5	11	*Baocun*(保存)	905	12149.6
2	*Diao*(掉)	5484	72882.2	12	*Pai*(拍)	1275	12124.9
3	*Yiliu*(遗留)	1541	26316.9	13	*Tui*(退)	1107	10914.4
4	*Sheng*(生)	2812	19081.8	14	*Baoliu*(保留)	784	9957.1
5	*Dun*(蹲)	1253	17883.1	15	*Na*(拿)	1121	9765.6
6	*Ta*(塌)	1098	16435.5	16	*Xie*(写)	1125	9711.1
7	*Jilu*(记录)	1285	16119.0	17	*Mai*(买)	1123	9303.5
8	*Shuai*(摔)	1186	15535.1	18	*Jianchi*(坚持)	959	8820.7
9	*Tiao*(跳)	1422	14915.9	19	*Queding*(确定)	775	7675.1
10	*Huo*(活)	1672	12835.5	20	*Xian*(闲)	641	7122.7

**Table 5:** Data list of 20 collocations with the strongest relevance to “V+ Xiaqu” construction.

Rank	Word	Frequency	collocational strength	Rank	Word	Frequency	collocational strength
1	*Zou*(走)	6909	70299.6	11	*Tun*(吞)	954	12045.0
2	*Huo*(活)	5405	54341.1	12	*Shengcun*(生存)	986	12021.5
3	*Jianchi*(坚持)	3759	44958.8	13	*Kan*(看)	1907	10600.3
4	*Shuo*(说)	5499	40405.3	14	*Cheng*(撑)	690	8283.8
5	*Jie*(接)	4054	39869.1	15	*Yanxu*(延续)	591	8192.5
6	*Tiao*(跳)	1809	19858.5	16	*Tuo*(拖)	764	8139.4
7	*Diao*(掉)	1602	17269.8	17	*Jinxing*(进行)	1099	7602.7
8	*Chi*(吃)	1814	13861.9	18	*Shuai*(摔)	565	6555.0
9	*He*(喝)	1354	12825.3	19	*Baochi*(保持)	699	6539.5
10	*Fazhan*(发展)	1783	12652.3	20	*Kaizhan*(开展)	728	6445.1

In terms of the number of types, “V+ *Xialai*” has the highest number of collocatable verbs (770), followed by “V+ *Xiaqu*” (711), and “V+ *Xia*” has the fewest (621). The order of the three constructions in terms of the number of verbs with an attraction relationship is consistent with the order of collocatable verb counts: “V+ *Xialai*” ranks the highest (554), “V+ *Xiaqu*” has 495, and “V+ *Xia*” is the lowest (396). To further assess collocational potential and productivity, we applied the type‑token ratio (TTR), first formally proposed by Chotlos for analyzing language samples [30]. TTR is calculated as the number of distinct verb types divided by the total number of tokens of that construction in the corpus; a higher TTR indicates greater lexical diversity. Two TTR values were computed for each construction, expressed in permillage (‰). The first, using all collocatable verb types, reflects collocational potential: “V+ *Xialai*” has the highest value (6.70‰), followed by “V+ *Xiaqu*” (6.20‰), and “V+ *Xia*” the lowest (0.73‰). The second, using only verbs with a significant attraction relationship, reflects the productivity of stable combinations: “V+ *Xialai*” again ranks highest (4.82‰), followed by “V+ *Xiaqu*” (4.31‰), and “V+ *Xia*” the lowest (0.46‰). A higher TTR indicates that a construction accommodates a wider range of verb types relative to its overall frequency, which is the operational definition of greater productivity. These results confirm that “V+ *Xialai*” has the greatest collocational diversity and productivity, while “V+ *Xia*” has the most restricted range of selectable verbs and the lowest productivity.

Prosodic requirements impose certain restrictions on the verb selection of “V+ *Xia*”. Prosody is part of the “macro-grammar” of Chinese: one syllable constitutes a rhythmic unit, and the evenly structured [1 + 1] disyllabic groups and [2 + 2] tetrasyllabic groups hold a dominant position in Chinese prosody [31]. Additionally, [2 + 1] structures forming compound nouns and [1 + 2] structures forming verb phrases are the prosodic norms of Chinese. Statistics show that there are only 7 disyllabic verbs (such as *Yiliu*(遗留) and *Baoliu*(保留))that have an attraction relationship with “V+ *Xia*”, accounting for 1.8%. All other verbs are monosyllabic. The difficulty in forming stable verb-direction constructions with disyllabic verbs limits the range of verb selection for “V+ *Xia*”. In contrast, there are 300 disyllabic verbs with an attraction relationship with “V+ *Xialai*”, accounting for 54.2%; and 225 disyllabic verbs with an attraction relationship with “V+ *Xiaqu*”, accounting for 45.5%. Benefiting from the dominant prosodic position of [2 + 2] tetrasyllabic groups, *Xialai* and *Xiaqu* have the potential to collocate with a wider range of verbs and are more likely to form stable combinations with disyllabic verbs. At the same time, since [1 + 2] structures forming verb phrases are a prosodic norm of Chinese, collocating with monosyllabic verbs does not become a prosodic obstacle for “V+ *Xialai*” and “V+ *Xiaqu*” to form verb-direction constructions. Specifically, there are 254 and 270 monosyllabic verbs that form an attraction relationship with these two constructions, respectively. In summary, “V+ *Xialai*” and “V+ *Xiaqu*” face fewer prosodic restrictions when forming verb-direction constructions compared to “V+ *Xia*”, resulting in higher collocational potential and productivity.

The average collocational strength of verbs with an attraction relationship to “V+ *Xia*” is 4597.0, while those for “V+ *Xialai*” and “V+ *Xiaqu*” are 1487.4 and 1377.1, respectively. Although “V+ *Xialai*” and “V+ *Xiaqu*” have more collocational combinations, their overall collocational strength values are lower—meaning the degree of collocational entrenchment is not as high as that of “V+ *Xia*”. Higher collocational strength reflects a tighter and more habitual association between a construction and its verb slots, which in usage‑based theory corresponds to greater cognitive entrenchment. Among the verbs with the highest correlation, the verb most strongly attracted to “V+ *Xia*” is *Ting*, with a strength value of 195602.9, which is higher than the peak values of “V+ *Xialai*” and “V+ *Xiaqu*”. The verb with the highest correlation to “V+ *Xialai*” is also *Ting*, but its collocational strength is lower at 150315.2. “V+ *Xiaqu*” has the lowest peak collocational strength: its most strongly attracted verb is *Zou*, with a collocational value of 70299.6. This shows that not only is the overall collocational strength of the “V+ *Xia*” construction higher, but the tightness and tendency of its high-frequency collocations are also stronger. The standard deviation of collocational strength values for all attraction-related verbs of “V+ *Xia*” reaches 14118.9, which is noticeably higher than those of “V+ *Xialai*” (7065.9) and “V+ *Xiaqu*” (5483.0). To determine whether the differences in means and standard deviations across the three constructions are statistically significant, we conducted permutation tests with 10,000 iterations. For the means, “V+ *Xia*” is significantly higher than both “V+ *Xialai*” and “V+ *Xiaqu*” (both p < 0.001, one‑tailed), while the difference between “V+ *Xialai*” and “V+ *Xiaqu*” is not significant (p = 0.790, two‑tailed). For the standard deviations, “V+ *Xia*” is significantly more dispersed than “V+ *Xiaqu*” (p = 0.0016, one‑tailed), but the difference between “V+ *Xia*” and “V+ *Xialai*” shows a marginal trend and does not reach statistical significance (p = 0.065, one‑tailed). Overall, “V+ *Xia*” exhibits significantly higher mean collocational strength than both “V+ *Xialai*” and “V+ *Xiaqu*”; its dispersion is significantly greater than that of “V+ *Xiaqu*”, while the comparison with “V+ *Xialai*” shows a marginal trend but does not reach statistical significance.

Lv and Liu have conducted in-depth research on the semantics and usage of “V+ *Xia*”, “V+ *Xialai*” and “V+ *Xiaqu*” [32–33]. Building on this foundation, this paper classifies the semantic types of the three verb-direction constructions, counts and annotates the selectable verbs for each semantic type. See Table 6.

**Table 6:** Semantic Types and Selectable Verbs of the Three Verb-Direction Constructions.

“V+ *Xia*”	Displacement from high to low	220 verbs (represented by *Zou*(走), *Chui*(垂), *Tiao*(跳), *Dun*(蹲))
Detachment	116 verbs (represented by *Ge*(割), *Chai*(拆), *Jian*(剪), *Yao*(咬))
Completion/Achievement	235 verbs (represented by *Xie*(写), *Zuo*(做), *Mai*(买), *Gong*(攻))
Contain	79 verbs (represented by *Zhuang*(装), *Zuo*(坐), *Rong*(容), *Zao*(造))
Transition from dynamic to static	27 verbs (represented by *Ting*(停), *Zhan*(站), *Li*(立), *Xie*(歇))
“V+ *Xialai*”	Displacement from high to low	291 verbs (represented by *Diao*(掉), *Ta*(塌), *Shuai*(摔), *Luo*(落))
	Detachment	122 verbs (represented by *Kan*(砍), *Huan*(换), *Ba*(拔), *Jie*(解))
	Completion/Achievement	358 verbs (represented by *Huo*(活), *Cun*(存), *Jilu*(记录), *Daying*(答应))
	State development	34 verbs (represented by *Leng*(冷), *Liang*(凉), *Andan*(暗淡), *Anding*(安定))
“V+ *Xiaqu*”	Displacement from high to low	223 verbs (represented by *Die*(跌), *Tun*(吞), *Ya*(压), *Hua*(滑))
	Detachment	70 verbs (represented by *Tui*(退), Tui(褪), *Cai*(裁), *shuai*(甩))
	Continuation of action	408 verbs (represented by *Shuo*(说), *Cheng*(撑), *Jianchi*(坚持), *Fazhan*(发展))
	State development	53 verbs (represented by *Shou*(瘦), *Re*(热), *Zui*(醉), *Weimi*(萎靡))

As noted at the beginning of this paper, all three constructions can express “displacement from high to low” (as illustrated in (1)). This semantic type belongs to the domain of spatial displacement, indicating that an object moves from a relatively higher position to a relatively lower one; it constitutes the basic spatial meaning shared by the three constructions. An examination of the semantic types of the three constructions reveals that they can all express detachment, i.e., the separation of a part from a whole through a specific action. For example:

**Table:** 

(4)	a.	把	这	块	木头	切下	。
		bǎ	zhè	kuài	mù tou	qiē xià	
	b.	P	this	piece	wood	cut off	
		把	这	块	木头	切下来	。
		bǎ	zhè	kuài	mù tou	qiē xià lái	
	c.	把	这	块	木头	切下去	。
		bǎ	zhè	kuài	mù tou	qiē xià qù	
		Cut this piece of wood off.					

The three examples in (4) all share the same core semantic content: separating a piece of wood from a larger whole through cutting. In this respect, their meanings are broadly similar. Nevertheless, the three constructions exhibit subtle differences in pragmatic orientation and cognitive reference. *Qiexia*(切下) focuses on an objective description of the cutting and separating action itself, merely indicating that a part is detached from the whole without presupposing any subsequent disposal of the detached part; it thus presents itself as a neutral depiction of the action. *Qiexialai*(切下来), in addition to marking the result of separation, carries a pragmatic implication that the detached part is to be obtained and retained for use, and the subsequent discourse may elaborate on what the cut-off piece of wood will be used for. In contrast, *Qiexiaqu*(切下去) conveys a pragmatic coloring of discarding or removing the separated part. These subtle functional distinctions enable speakers to make contextually appropriate choices among the three constructions.

Both “V+ *Xia*” and “V+ *Xialai*” can express completion/achievement. The two constructions are semantically close and largely interchangeable in many contexts. “V+ *Xia*” carries a stronger transitive and dispositional force, focusing on the attainment of a goal or the realization of a result (i.e., achievement), whereas “V+ *Xialai*” places greater emphasis on the completion or finalization of the event as a whole, highlighting that the action is brought to a close and the resultant state is secured. The core difference between the two constructions lies in their syntactic behavior: “V+ *Xia*” permits a postverbal patient object, whereas “V+ *Xialai*” does not; to co-occur with a patient, “V+ *Xialai*” requires the patient to be fronted by a preposition and placed before the construction, as illustrated in (5):

**Table:** 

(5)	a.	他	决定	买下	那	房子	。	
		tā	jué dìng	mǎi xià	nà	fáng zi		
		3SG	decide	buy	that	house		
	b.	他	决定	把	那	房子	买下来	。
		tā	jué dìng	bǎ	nà	fáng zi	mǎi xià lái	
		3SG	decide	P	that	house	buy	
		He decided to buy that house.						

In (5a), *Maixia*(买下) can be directly followed by the patient object *Fangzi*(房子). By contrast, *Maixialai*(买下来) in (5b) cannot take a postverbal patient(“*他决定买下来房子”); if it is to co-occur with a patient argument, the patient must be introduced by a preposition such as *Ba*(把) and placed before the verb-direction construction.

Both “V+ *Xialai*” and “V+ *Xiaqu*” can express state development, yet they differ in semantic orientation. “V+ *Xialai*” typically encodes gradable perceptual properties, such as temperature, light, color, atmosphere, or emotion, indicating that a state changes gradually and settles into a perceptible resultant state. “V+ *Xiaqu*”, by contrast, emphasizes the sustained extension of a state over time. For example:

**Table:** 

(6)	a.	天空	渐渐	暗淡下来	。
		tiān kōng	jiàn jiàn	àn dàn xià lái	
		the sky	gradually	darken	
		The sky gradually darkened.			
		老人	一天天	消瘦下去	。
		lǎo rén	yì tiān tiān	xiāo shòu xià qù	
		old man	day by day	waste away	
		The old man was wasting away with each passing day.			

In addition to the shared semantic types discussed above, “V+ *Xia*” and “V+ *Xiaqu*” exhibit distinct semantic types that are not typically available to the others. “V+ *Xia*” can express contain, indicating the capacity to hold or accommodate entities within a spatial or abstract boundary, as well as transition from dynamic to static, denoting the shift of an action or a subject from a state of motion or activity to a state of relative stillness or stability. “V+ *Xiaqu*” is uniquely specialized in expressing continuation of action, wherein an action or process is sustained and extended along the temporal axis. These three distinct semantic types are illustrated in (7) through (9):

**Table:** 

(7)	会议室	能	坐下	这么多	人	吗	？	
	huì yì shì	néng	zuò xià	zhè me duō	rén	ma		
	conference room	can	accommodate	so much	people	Q		
	Can the meeting room seat this many people?							
(8)	站下	，	先	别	急着	走	！	
	zhàn xià		xiān	bié	jí zhe	zǒu		
	Stop walking		first	don’t	rush to	leave		
	Stop (and stand still), don’t be in a hurry to leave!							
(9)	这个	开头	说	得	不错	，	说下去	。
	zhè ge	kāi tóu	shuō	de	bú cuò		shuō xià qù	
	this	begining	speak	DER	well		go on speaking	
	You’ve started well. Go on speaking.							

**Fig 4:**
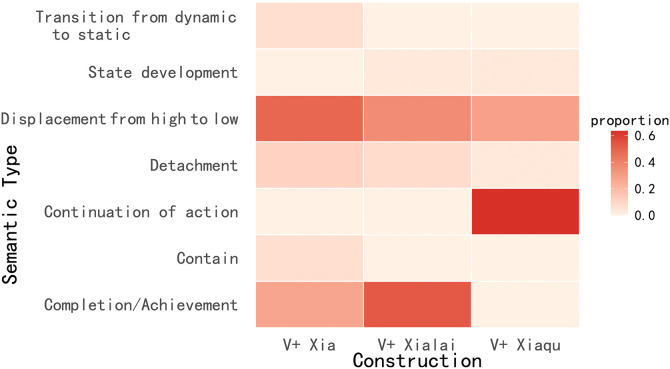
The collocational strength distribution of the semantic types of “V+ *Xia*”, “V+ *Xialai*”, and “V+ *Xiaqu*”.

Some verb-direction constructions formed by specific verbs have multiple semantic types. In particular, most collocations of verbs with “V+ *Xiaqu*” can express the meaning of “continuation of action”. For example, *Tiao Xiaqu*(跳下去) mainly conveys the meaning of “displacement from high to low” (e.g., “从台阶上跳下去”), but it can also express “continuation of action” in certain contexts (e.g., “再这么跳下去，膝盖就伤了”). Therefore, when calculating the collocational strength between these verbs and each semantic type of the verb-direction construction, it is necessary to allocate the strength proportionally. The distribution of collocational strength across different semantic types of “V+ *Xia*”, “V+ *Xialai*”, and “V+ *Xiaqu*” is shown in Fig 4.

The constructional meaning of a construction is manifested through its usage instances. A high-frequency usage instance of a construction usually becomes the prototype of that construction, promoting the establishment and entrenchment of the construction’s form-meaning rules [34]. The prototype meaning is the most typical and representative core meaning of a polysemous construction, and it is the most prominent and first thought-of meaning when people cognize the construction. As shown in Fig 4, the semantic type with the highest collocational strength for “V+ *Xia*” is “displacement from high to low”, accounting for 48.3%. Within a usage-based framework, the identification of prototype meaning is grounded in usage patterns attested in authentic corpus data: the semantic type that co-occurs with the construction most frequently and with the greatest collocational strength constitutes the most cognitively entrenched meaning, and thus the prototype meaning of the construction. Therefore, “displacement from high to low” can be regarded as the prototype meaning of “V+ *Xia*”, while other semantic types occupy a subordinate position in the construction family. Although this semantic type is also prominent in “V+ *Xialai*” and “V+ *Xiaqu*”, with a non-negligible distribution proportion, it is not the most frequent one in the two construction families, so it is not their prototype meaning. The semantic type with the highest collocational strength for “V+ *Xialai*” is “completion/achievement”, while that for “V+ *Xiaqu*” is “continuation of action”. That is, when Chinese speakers cognize “V+ *Xialai*” and “V+ *Xiaqu*”, they will first or most easily understand them as “completing something/achieving a certain standard” and “continuation of an action”, respectively. This is also consistent with the distribution of verb selection for the two constructions: 358 verbs can collocate with *Xialai* to express “completion/achievement”, accounting for 64.6% of the total number of attraction-related verbs in “V+ *Xialai*” (554 verbs); 408 verbs can collocate with *Xiaqu* to express “continuation of action”, accounting for 82.4% of the total number of attraction-related verbs in “V+ *Xiaqu*” (495 verbs).

The “V+ *Xia*” construction has more semantic types than “V+ *Xialai*” and “V+ *Xiaqu*”. Its collocational strength is more evenly distributed across types, and the proportion of collocational strength accounted for by non-prototype meanings is also higher. In contrast, “V+ *Xialai*” and “V+ *Xiaqu*” have fewer semantic types, and their collocational strength is more concentrated on their prototype meanings—accounting for 52.7% and 63.6% respectively. Notably, spatial displacement is not the core meaning of these two constructions.

### Analysis of collocational tendencies of high-frequency lexical items across the constructions “V+ *Xia*”, “V+ *Xialai*”, and “V+ *Xiaqu*”

In linguistics, discussions on classifying verbs based on the criterion of aktionsart (action type) have a long history and yielded abundant results. Building on the research findings of Smith, Olsen, Zuo, and others, this study classifies verbs into 7 categories using four semantic features: [dynamic], [sustainable], [quantitative change], and [attribute]. Smith used [dynamic] as a criterion to identify “activity verbs” (which emphasize action) and [durative] to distinguish “non-instantaneous verbs” [35]. Considering that some verbs in this latter category can continue through repetition (e.g., *Kesou*(咳嗽, cough) and *Qiao*(敲, knock) can both be sustained), this study adopts [sustainable] as the classification criterion instead. Olsen proposed the category of “stage-level state” verbs, which includes verbs with the feature of “gradual change in degree or quantity” (i.e., [quantitative change]), such as *Huaiyun*(怀孕, be pregnant) [36]. Zuo argued that there are a considerable number of “dual-character verbs” in Chinese and classified them into two subcategories: verbs that concurrently express “activity and result”, and verbs that concurrently express “activity and state” [37]. He also advocated that qualitative adjectives capable of functioning independently as predicates (e.g., *Hao* (好), *Da* (大), *Zhong*(重)) can be treated as verbs, and these adjectives are closely related to psychological verbs. This study uses the [attribute] feature to distinguish between the two: qualitative adjectives that describe the attributes and characteristics of things have the [+attribute] semantic feature, while psychological verbs do not. The specific classification is shown in Table 7.

**Table 7:** Verb Classification and Semantic Features of Each Category.

Verb Category	[Dynamic]		[Sustainable]	[Quantitative Change]		[Attribute]
Strong Activity Verbs (e.g., *Pao* (跑), *Tiao* (跳)	**+**		**+**	**–**		**–**
Weak Activity Verbs (e.g., *Zuo* (做), *Mai* (买)	**–**		**+**	**–**		**–**
Psychological Verbs (e.g., *Ai*(爱), *Yuan* (怨)	**–**		**+**	**+**		**–**
Qualitative Adjectives (e.g., *Hao* (好), *Da* (大)	**–**		**+**	**+**		**+**
Relational Verbs (e.g., *Shi* (是), *Shuyu* (属于)	**–**		**+**	**–**		**+**
Activity-Result Verbs (e.g., *Yu* (遇), *Jiao*(交)	**+**	**–**	**–**	**–**		**–**
Activity-State Verbs (e.g., *Si* (死), *Fu* (浮)	**+**	**–**	**+**	**+**	**–**	**–**

**Fig 5:**
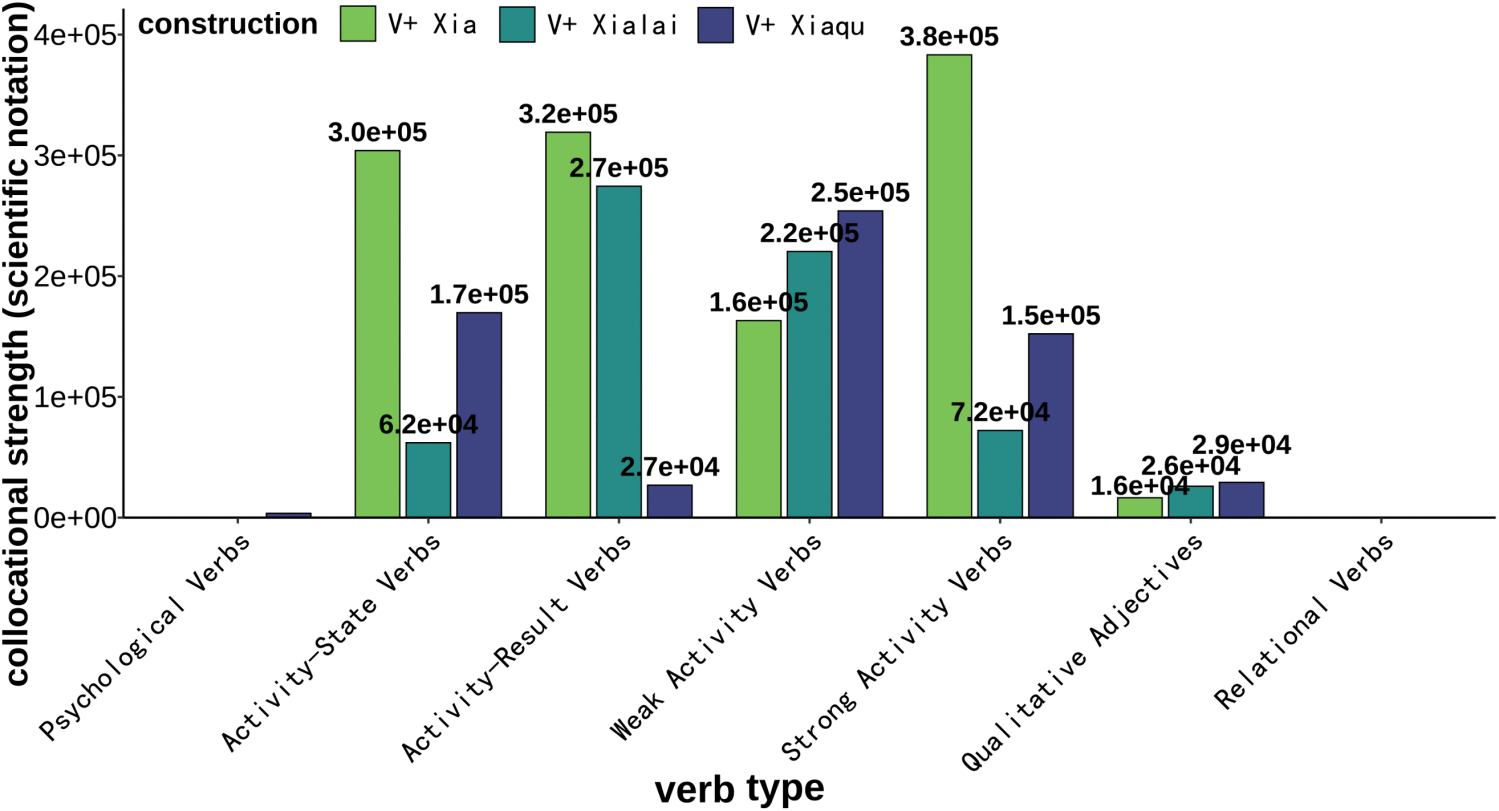
Comparison of Collocational Strength Between Each Verb Category and the Verb-Direction Constructions “V+ *Xia*”, “V+ *Xialai*”, and “V+ *Xiaqu*”.

By applying the collexeme analysis method, the collocational strength between each verb category and the verb-direction constructions “V+ *Xia*”, “V+ *Xialai*”, and “V+ *Xiaqu*” can be calculated. See Fig 5.

The differences in the prototype meanings of “V+ *Xia*”, “V+ *Xialai*”, and “V+ *Xiaqu*” lead to obvious differences in their tendencies toward different verb categories. As shown in Fig 5, “V+ *Xia*”, whose prototype meaning is “spatial movement (displacement from high to low)”, has the highest tendency toward strong activity verbs (which emphasize action), and this tendency is much higher than that of “V+ *Xialai*” and “V+ *Xiaqu*”. Additionally, “V+ *Xia*” also has higher collocational strength with the two verb categories of “activity-result verbs” and “activity-state verbs” compared to “V+ *Xialai*” and “V+ *Xiaqu*”. “V+ *Xia*” shows excellent compatibility with verbs carrying the [+dynamic] semantic feature, but its attraction to [-dynamic] verbs is weaker than that of “V+ *Xialai*” and “V+ *Xiaqu*”. Among the verbs with an attraction relationship to “V+ *Xia*”, there are no psychological verbs; moreover, its collocational strength with weak activity verbs and qualitative adjectives is the lowest among the three constructions. “V+ *Xiaqu*”, whose prototype meaning is “continuation of action”, has a high degree of association with verbs carrying the [+sustainable] feature. It is not only the only one among the three constructions that shows significant attraction to psychological verbs, but also has the highest collocational strength with weak activity verbs and qualitative adjectives. Furthermore, its attraction to activity-state verbs is significantly higher than that of “V+ *Xialai*”. “V+ *Xialai*” does not have the highest collocational strength with any single verb category. However, as a construction with “completion/achievement” as its prototype meaning, it has high attraction to activity-result verbs: its collocational strength in this category is slightly lower than that of “V+ *Xia*”, but much higher than that of “V+ *Xiaqu*”.

In addition to the macro-level comparison of differences in tendencies toward different verb categories, differences in high-frequency usage instances can reflect more specific features of the constructions. We conducted distinctive collexeme analysis on the high-frequency collocational words of “V+ *Xia*”, “V+ *Xialai*”, and “V+ *Xiaqu*” in pairs. Tables 8–10 list 10 representative pairs of contrastive high-frequency words.

**Table 10:** Distinctive Collexeme Analysis of “V+ Xialai” and “V+ Xiaqu”.

Collocational Word	Frequency in “V+ *Xialai*”	Frequency in “V+ *Xiaqu*”	Log-Odds-Ratio	Collocational Tendency
*Jilu*(记录)	1285	0	Inf	“V+ *Xialai*”
*Gong*(攻)	80	0	Inf	“V+ *Xialai*”
*Shuai*(摔)	1186	565	0.74	“V+ *Xialai*”
*Xie*(写)	1125	654	0.54	“V+ *Xialai*”
*Hua*(滑)	555	424	0.27	“V+ *Xialai*”
*Tiao*(跳)	1422	1809	−0.25	“V+ *Xiaqu*”
*Jianchi*(坚持)	959	3759	−1.39	“V+ *Xiaqu*”
*Tuo*(拖)	183	764	−1.44	“V+ *Xiaqu*”
*Zou*(走)	1059	6909	−1.93	“V+ *Xiaqu*”
*Chi*(吃)	93	1814	−2.99	“V+ *Xiaqu*”

**Table 8:** Distinctive Collexeme Analysis of “V+ Xia” and “V+ Xialai”.

Collocational Word	Frequency in “V+ *Xia*”	Frequency in “V+ *Xialai*”	Log-Odds-Ratio	Collocational Tendency
*Chi*(吃)	3367	93	1.59	“V+ *Xia*”
*Gong*(攻)	1376	80	0.84	“V+ *Xia*”
*Zuo*(做)	2043	134	0.72	“V+ *Xia*”
*Chui*(垂)	5965	576	0.33	“V+ *Xia*”
*Zou*(走)	10571	1058	0.30	“V+ *Xia*”
*Na*(拿)	7822	1121	−0.07	“V+ *Xialai*”
*Tiao*(跳)	7017	1422	−0.42	“V+ *Xialai*”
*Hua*(滑)	2061	555	−0.70	“V+ *Xialai*”
*Bei*(背)	583	462	−1.78	“V+ *Xialai*”
*Jilu*(记录)	0	1285	-Inf	“V+ *Xialai*”

**Table 9:** Distinctive Collexeme Analysis of “V+ Xia” and “V+ Xiaqu”.

Collocational Word	Frequency in “V+ *Xia*”	Frequency in “V+ *Xiaqu*”	Log-Odds-Ratio	Collocational Tendency
*Gong*(攻)	1376	0	Inf	“V+ *Xia*”
*Mai*(买)	4939	30	3.10	“V+ *Xia*”
*Na*(拿)	7822	74	2.66	“V+ *Xia*”
*Bei*(背)	583	40	0.67	“V+ *Xia*”
*Xie*(写)	8743	654	0.59	“V+ *Xia*”
*Hua*(滑)	2061	424	−0.43	“V+ *Xiaqu*”
*Tiao*(跳)	7017	1809	−0.66	“V+ *Xiaqu*”
*Chi*(吃)	3367	1814	−1.40	“V+ *Xiaqu*”
*Zou*(走)	10571	6909	−1.63	“V+ *Xiaqu*”
*Jianchi*(坚持)	0	3759	-Inf	“V+ *Xiaqu*”

The p-values from Fisher’s exact test for the above distinctive collexeme analysis results are all less than 0.05, indicating that the conclusions about collocational tendencies are reliable. The log-odds-ratio is an effect size indicator that reflects the preference tendency and degree of a collocational word toward two constructions. A positive value means the word prefers the first-mentioned construction, while a negative value means it prefers the second-mentioned one; the larger the absolute value, the stronger the preference. By comparing the high-frequency lexical items with strong collocational tendencies across the three constructions, the following can be observed: Due to the differences in their prototype meanings and the significant variations in the distribution of collocational strength across semantic types, many verbs that can only collocate with a specific construction to express its unique semantic meaning show clear preferences. When compared with other constructions, these verbs have a larger absolute value of log-odds-ratio. However, when expressing spatial displacement, the three constructions compete fiercely for attracting verbs: the verbs’ tendencies toward specific constructions are relatively vague, and thus the absolute values of their log-odds-ratios are relatively lower.

Both “V+ *Xia*” and “V+ *Xialai*” can express “completion/achievement”, but they have different semantic emphases. The “completion/achievement” meaning of “V+ *Xia*” has historically derived from the concrete meaning of *Xia* as “conquer” or “take (a place)”, an extension attested as early as the Han Dynasty [38]. Consequently, “V+ *Xia*” shows a stronger attraction than “V+ *Xialai*” to verbs with a “conquering” semantic tendency, such as *Gong*(攻, attack) and *Da*(打, fight). “V+ *Xialai*” takes “completion/achievement” as its prototype meaning and can express completion in a general sense; it has a stronger collocational tendency toward most verbs that can combine with *Xialai* to express completion, including *Na*(拿), *Bei*(背), *Huo*(活), *Pai*(拍). *Xie*(写, write) is a verb with high usage frequency in Chinese; it has significant attraction and high collocational strength with both “V+ *Xia*” and “V+ *Xialai*”. However, the test p-value between the two constructions is greater than 0.05, which means *Xie* has no significant tendency toward either “V+ *Xia*” or “V+ *Xialai*”. The attraction of the two constructions to *Xie* is almost the same, so the choice between them in use depends more on contextual judgment.

When expressing spatial displacement, “V+ *Xia*”, “V+ *Xialai*”, and “V+ *Xiaqu*” can all convey displacement from high to low, with the main difference lying in the reference point used to judge the direction of the displacement movement. The reference point for “V+ *Xia*” is usually a scene-default reference point, taking other relatively stationary entities in the same spatial domain or scene as the reference, and the observer or speaker describes the displacement event from high to low from a bystander’s perspective. “V+ *Xialai*” and “V+ *Xiaqu*” more often adopt an observer-based reference point, taking the observer or speaker themselves as the reference and perceiving the distance between the displaced object and themselves as well as their relative interactive relationship from an experiencer’s perspective. Additionally, in use, “V+ *Xialai*” and “V+ *Xiaqu*” can set temporary reference points based on subjective viewpoints and emotional stances to highlight the approach/away relationship between the displaced object and the viewpoint position/object. For example, “把东西给您拿下来” (“bring the things down for you”) sets the temporary reference point on “您” (Honorific name of “you”), emphasizing the approach of “the things” to “you”.

The reference points chosen by “V+ *Xialai*” and “V+ *Xiaqu*” are closely related to the interactive relationship between the displaced object and the target. Therefore, when the displacement event expressed by a verb emphasizes the interaction between the displaced object and the observer or viewpoint object, the verb is likely to show a stronger tendency toward “V+ *Xialai*” and “V+ *Xiaqu*” than toward “V+ *Xia*”. Verbs such as *Tiao* (跳, jump), *Hua* (滑, slide), *Diao* (掉, fall), *Shuai* (摔, fall down), and *Tui* (退, retreat) all have a higher tendency toward “V+ *Xialai*” and “V+ *Xiaqu*” than toward “V+ *Xia*”. This means that when Chinese speakers cognize these verbs, they mostly perceive the interaction between the displaced object and themselves or the viewpoint object from an experiencer’s perspective. In contrast, verbs like *Chui*(垂, hang down), *Die* (跌, fall), and *Sa* (洒, sprinkle) are more inclined toward “V+ *Xia*”, which indicates that when cognizing the displacement events they represent, people mostly adopt a bystander’s perspective.

By referring to Table 10, we can determine whether verbs such as *Tiao*(跳), *Hua* (滑), *Diao* (掉), *Shuai* (摔), and *Tui* (退)—which all show a higher tendency toward “V+ *Xialai*” and “V+ *Xiaqu*” than toward “V+ *Xia*”—are more inclined to express “approach” or “away”. The data shows that *Hua* (滑), *Shuai*(摔), *Diao* (掉), and *Tui* (退) are more inclined to express “approach”, while *Tiao*(跳) is more inclined to express “away”. By referring to Tables 8 and 9, we can find that *Zou*(走) has the strongest tendency toward “V+ *Xiaqu*”, and its tendency toward “V+ *Xia*” is higher than that toward “V+ *Xialai*”. However, the significant tendency of verbs like *Zou*, *Tiao*, and *Pao*(跑) toward “V+ *Xiaqu*” is influenced by the large number of corpus examples where these verbs collocate with *Xiaqu* to express “continuation of action”. To determine their tendency toward displacement direction, it is necessary to exclude these relevant examples before calculation. After comparison, it is found that *Tiao* still shows the strongest tendency toward “V+ *Xiaqu*”, while *Zou* and *Pao* show the strongest tendency toward the verb-direction construction “V+ *Xia*” when expressing spatial displacement.

In addition to differences in reference point selection and displacement direction expression, *Lai* and *Qu* add a “completion” meaning to “V+ *Xialai*” and “V+ *Xiaqu*” when these two constructions express spatial displacement. Cao argues that the directional verb *Lai* indicates that an object moves toward the speaker’s location; when this movement extends from space to time, if the start of an action is taken as the starting point and the end as the finishing point, it will develop a usage that expresses “completion”. The directional verb *Qu* indicates that an object moves toward a target; when this movement shifts from moving toward a spatial target to moving toward a temporal or situational target, it refers to “what is about to happen” or “what is about to be completed or realized”. When the restriction of “about to” is removed, it becomes a general expression of “completion” or “realization”. The “completion” meanings of *Lai* and *Qu* are integrated into “V+ *Xialai*” and “V+ *Xiaqu*” when they form these constructions. Therefore, even for the same verb that shows significant attraction to all three constructions, “V+ *Xialai*” and “V+ *Xiaqu*” have more semantics than “V+ *Xia*” to express “complete the whole displacement event” when expressing spatial displacement. This difference leads to variations in the usage scenarios of the three constructions: “V+ *Xia*” can be used during the object’s displacement process to describe the object’s immediate movement state; “V+ *Xialai*” and “V+ *Xiaqu*” are usually used either when the event has already occurred to indicate the completion of displacement or when the event has not yet occurred to indicate that displacement is about to be completed.

### Multidimensional scaling analysis of “V+ *Xia*”, “V+ *Xialai*”, and “V+ *Xiaqu*”

**Fig 6:**
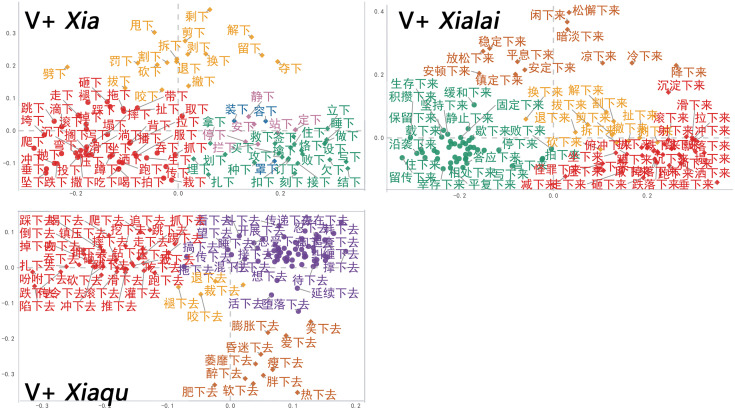
Multidimensional Scale Analysis of the Verb-Direction Constructions “V+ *Xia*”, “V+ *Xialai*”, and “V+ *Xiaqu*”.

In the previous sections, we completed a quantitative comparison of the collocational strength between the verb-direction constructions “V+ *Xia*”, “V+ *Xialai*”, and “V+ *Xiaqu*” (and their respective semantic types) and their selectable verbs, as well as the collocational tendencies of different verb types across the constructions. Building on this foundation, we can conduct a Multidimensional Scaling (MDS) analysis of the three constructions. First, we map the semantic information of the verb-direction constructions to high-dimensional numerical vectors. Then, we use the Jensen-Shannon Divergence (JSD) to measure the differences in semantic distribution between the constructions. Finally, we map the high-dimensional distances to a two-dimensional plane through eigenvalue decomposition, presenting the relative distance relationships between the constructions as a semantic network structure. See Fig 6.

As shown in Fig 6, in the MDS plot of the three constructions, circular scatter points represent the distribution of the constructions’ prototype meanings. The colors of these points differ because the prototype meanings of the three constructions are distinct. Diamond scatter points represent other subordinate semantic types of the constructions, and the distance between scatter points reflects the relative semantic distance between the constructions. Visual inspection suggests that the prototype distributions are unimodal for all three constructions, with scatter points tightly clustered around a single central region without splitting into distinct subgroups. This supports the interpretation that each construction has one coherent and singular prototype meaning, and that the core categories of the three constructions have strong semantic consistency. In contrast, non‑prototype meanings are more widely scattered in the edge areas, indicating greater diversity among subordinate semantic types.

By comparing the semantic networks of “V+ *Xia*”, “V+ *Xialai*”, and “V+ *Xiaqu*”, the following can be observed: The semantic networks of “V+ *Xia*” and “V+ *Xiaqu*” exhibit a gradient feature, meaning the constructions are distributed continuously from prototype meanings to non-prototype meanings without clear boundaries or gaps. The distance between each semantic type is relatively short. In contrast, the distance between the non-prototype meanings and prototype meaning of “V+ *Xialai*” is relatively long, and its “state development” meaning shows a tendency to form an independent cluster. While “V+ *Xia*” and “V+ *Xiaqu*” are overall continuous, they still have differences: The prototype meaning of “V+ *Xia*” is “displacement from high to low”, which is only adjacent to the “completion/achievement” meaning and “detachment” meaning in distribution. The “containment” meaning and “transition from dynamic to static” meaning are far from the prototype meaning; instead, they overlap with the distribution area of the “completion/achievement” meaning, and many constructions have two or even three of these semantic meanings concurrently. For “V+ *Xiaqu*”, all non-prototype meanings are clearly distributed continuously around its prototype meaning, and the “continuation of action” meaning serves as the core of its semantic network.

Regarding the distribution of non-prototype meanings across the three constructions, for semantic types with relatively high collocational strength, such as the “completion/achievement” meaning of “V+ *Xia*” and the “displacement from high to low” meaning of “V+ *Xialai*” and “V+ *Xiaqu*”. the distance between different constructions within the same semantic type is relatively short, indicating high semantic consistency. In contrast, for some semantic types with relatively low collocational strength such as the “detachment” meaning of “V+ *Xia*” and the “state development” meaning of “V+ *Xialai*” and “V+ *Xiaqu*”, the distance between constructions is relatively long and their distribution is scattered. This shows that these semantic types have the characteristic of diversity.

### Robustness check

To ensure that the collocational strength reported in this study, together with the conclusions based on it, is not systematically confounded by verb lemma frequency, we further performed a robustness check using mixed‑effects models. The concept of mixed‑effects models can be traced back to Fisher’s work on variance components and random effects [39], and was subsequently developed by scholars such as Henderson and Harville [40–42]. Laird & Ware provided a systematic framework for linear mixed‑effects models in longitudinal data analysis, thereby facilitating their application in empirical research [43]. Baayen played a central role in systematically introducing mixed‑effects models into linguistics and promoting their widespread adoption [44]. Gries further demonstrated and refined the applicability and analytical procedures of this method in corpus linguistics [45–46].

The specific modeling steps are as follows. For each construction (“V+ *Xia*”, “V+ *Xialai*”, “V+ *Xiaqu*”), we constructed a verb‑construction‑level dataset, in which each row records the frequency of a given verb in that construction (*freq*_const_) and its lemma frequency in the BCC corpus (*freq*_lemma_). We employed a generalized linear mixed model (GLMM) with aggregated binomial specification. The dependent variable is a binomial count vector, with the verb’s frequency in the target construction defined as “success” (*freq*_const_) and its frequency outside that construction as “failure” (*freq*_lemma_- *freq*_const_), forming the binomial pair cbind(*freq*_const_, *freq*_lemma_ – *freq*_const_). As the sole fixed effect, we included the natural logarithm of the verb’s lemma frequency (log(*freq*_lemma_)). The purpose is to control for the log‑linear effect of verb lemma frequency on the dependent variable, thereby mitigating the potential confounding effect of the verb’s baseline usage frequency. For the random‑effect part, we included a random intercept for verb lemma ((1 | verb)), which captures overdispersion at the verb level, i.e., the heterogeneity among verbs that exceeds the expectation of the binomial distribution. After model estimation, the random intercept for each verb reflects its residual attraction to the construction after controlling for lemma frequency — that is, the residual association strength above and beyond what is expected from frequency alone. Subsequently, we computed Spearman’s rank correlation coefficient ρ between the Fisher‑based collocational strength ranking and the random‑intercept ranking for each construction.

The Spearman’s rank correlation coefficient is a nonparametric statistic that measures the degree of consistency between two rank sequences. It concerns only the relative order of the data and does not require the variables to follow a normal distribution or a linear relationship, making it particularly suitable for comparing the rankings produced by different methods on the same set of objects. The value of ρ ranges from −1 to +1: ρ close to +1 indicates perfect agreement between the two rankings, ρ close to 0 indicates no monotonic correlation, and ρ close to −1 indicates perfect inverse agreement. In fields such as corpus linguistics and computational linguistics, ρ ≥ 0.80 is commonly considered a strong correlation, and ρ ≥ 0.90 a very strong correlation. The Spearman’s rank correlation coefficients are reported in Table 11.

**Table 11:** Spearman’s rank correlation coefficients of “V+ Xia”, “V+ Xialai”, and “V+ Xiaqu”.

Construction	Spearman’s ρ	p‑value
V + *Xia*	0.978	< 0.001
V+ *Xialai*	0.987	< 0.001
V+ *Xiaqu*	0.988	< 0.001

The results above show that the ρ values for all three constructions are well above the 0.90 threshold for a “very strong correlation”, and the p‑values are all below 0.001, indicating that the consistency between the Fisher‑based collocational strength ranking and the mixed‑effects model random‑intercept ranking is highly statistically significant. This means that the ordering of verbs by their attraction to each construction remains largely unchanged even after rigorous control for verb lemma frequency. Table 12 illustrates the adjustment of residual attraction for 30 representative verbs after controlling for verb lemma frequency using the mixed‑effects model (full random‑intercept results for all attracted verbs are provided in Appendix 4).

**Table 12:** Random intercepts of representative verbs for the three constructions.

Verb	Construction	Random Intercept	Collocational Strength
*Ting*(停)	V + *Xia*	4.04	195602.94
*Zou*(走)	V + *Xia*	3.13	73783.08
*Jian*(剪)	V + *Xia*	1.39	7190.48
*Zhong*(种)	V + *Xia*	1.04	3984.18
*Zhuai*(拽)	V + *Xia*	0.10	1277.12
*Bai*(掰)	V + *Xia*	−0.15	906.45
*Cai*(采)	V + *Xia*	−0.65	197.60
*Sai*(塞)	V + *Xia*	−1.20	171.66
*Fang*(防)	V + *Xia*	−1.75	4.60
*Qu*(屈)	V + *Xia*	−2.74	7.46
*Ting*(停)	V+ *Xialai*	5.01	137543.47
*Jilu*(记录)	V+ *Xialai*	3.22	16118.98
*Zou*(走)	V+ *Xialai*	2.48	6754.36
*Piao*(飘)	V+ *Xialai*	1.12	1342.29
*Chenmo*(沉默)	V+ *Xialai*	0.28	475.38
*Bo*(拨)	V+ *Xialai*	−0.15	260.34
*Jiejiu*(解救)	V+ *Xialai*	−0.50	182.90
*Sao*(扫)	V+ *Xialai*	−0.84	92.83
*Ta*(拓)	V+ *Xialai*	−1.44	36.73
*Yin*(引)	V+ *Xialai*	−1.77	2.18
*Zou*(走)	V+ *Xiaqu*	4.04	70299.58
*Shuo*(说)	V+ *Xiaqu*	3.43	40405.26
*Jiangchi*(僵持)	V+ *Xiaqu*	1.76	1285.18
*Wa*(挖)	V+ *Xiaqu*	0.90	935.27
*Shou*(守)	V+ *Xiaqu*	0.08	333.87
*Ku*(哭)	V+ *Xiaqu*	−0.05	292.81
*Diaocha*(调查)	V+ *Xiaqu*	−1.11	51.86
*Langfei*(浪费)	V+ *Xiaqu*	−1.29	42.90
*Yin*(饮)	V+ *Xiaqu*	−1.63	20.55
*Xiu*(修)	V+ *Xiaqu*	−1.94	6.10

The random intercept provides a continuous measure of each verb’s attraction to the construction after controlling for verb lemma frequency. A positive random intercept indicates that the verb’s residual attraction is above the mean level of all attracted verbs for that construction, whereas a negative value indicates a below‑mean level. As can be seen from Table 12, within each construction the ordering of verbs by random intercept is largely consistent with their ordering by collocational strength: verbs with higher collocational strength generally also have higher random intercept values. For example, *Ting* and *Zou* in “V+ *Xia*”, *Ting* and *Jilu* in “V+ *Xialai*”, and *Zou* and *Shuo* in “V+ *Xiaqu*” rank at the top on both measures, which further confirms the reliability of our collocational strength calculation. Verbs with negative random intercepts are typically located at the lower end of the collocational strength ranking. Among these, verbs with large absolute values, such as *Qu* and *Fang* in “V+ *Xia*”, *Yin* and *Ta* in “V+ *Xialai*”, and *Xiu* and *Yin* in “V+ *Xiaqu*”, exhibit attraction that is largely driven by the statistical effect of their low lemma frequency. Nevertheless, the Spearman’s rank correlation coefficients for the three constructions all exceed 0.97, and such verbs remain significantly positive in the FDR‑corrected Fisher test. This indicates that verbs with negative random intercepts are merely located near the tail of the “significantly attracted” group and do not affect the main conclusions of this study. Consequently, the core findings reported in this study, including the collocational strength rankings, the identification of prototype meanings, and the distribution of semantic types, are highly robust.

## Conclusions

Based on construction grammar and prototype theory, this study adopts the collostructional analysis method to quantitatively analyze and compare the collocational strength between the modern Chinese verb-direction constructions “V+ *Xia*”, “V+ *Xialai*”, “V+ *Xiaqu*” and their selectable verbs. The findings are as follows: Among the three constructions, “V+ *Xialai*” can collocate with the largest number of verbs, while “V+ *Xia*” can collocate with the smallest number. Phonological constraints result in the narrowest collocation range and the lowest productivity of “V+ *Xia*”. However, the collocational strength of verbs with an attraction relationship to “V+ *Xia*” is generally higher than that of verbs related to “V+ *Xialai*” and “V+ *Xiaqu*”; additionally, the tightness and tendency of its high-frequency collocations are also stronger. The three constructions have different prototype meanings: the prototype meaning of “V+ *Xia*” is “displacement from high to low”, that of “V+ *Xialai*” is “completion/achievement”, and the core meaning of “V+ *Xiaqu*” is “continuation of action”. “V+ *Xia*” has more semantic types than “V+ *Xialai*” and “V+ *Xiaqu*”, with a higher proportion of collocational strength accounted for by non-prototype meanings and a more even overall distribution. In contrast, “V+ *Xialai*” and “V+ *Xiaqu*” have relatively fewer semantic types, and their collocational strength is more concentrated on their prototype meanings. Differences in prototype meanings lead to significant variations in the types of verbs attracted by the three constructions. However, when expressing spatial displacement, they compete fiercely, resulting in vague verb tendencies. The semantic networks of “V+ *Xia*” and “V+ *Xiaqu*” generally exhibit a gradient feature, while the “state development” meaning of “V+ *Xialai*” shows a tendency to form an independent cluster. The prototype meanings of the three constructions have strong semantic consistency: semantic types with higher collocational strength show stronger consistency in distribution, while some semantic types with lower collocational strength are distributed discretely and exhibit diversity. Further work could extend the present findings by examining the behavior of these verb‑direction constructions across different registers in a more fine‑grained manner and systematically calculating dispersion across texts.
