# Correction: Quantitative comparative study on the verb-direction constructions “V+ *Xia* (下)”, “V+ *Xialai* (下来)” and “V+ *Xiaqu* (下去)” in mandarin Chinese

**DOI:** 10.1371/journal.pone.0357386

**Published:** 2026-08-31

**Authors:** Tian Tan, Jiangcai Li

There is an error in affiliation 2 for author Jiangcai Li. The correct affiliation 2 is: College of Liberal Arts, Hunan Normal University, Changsha, Hunan, China.
