# Supplementary material for: Quantitative comparative study on the verb-direction constructions “V+ Xia (下)”, “V+ Xialai (下来)” and “V+ Xiaqu (下去)” in mandarin Chinese
Source: PLoS One. 2026 Jul 29;21(7):e0354793. doi: 10.1371/journal.pone.0354793 (PMC13419241; doi:10.1371/journal.pone.0354793)
Supplement: Attachment — Submitted filename: Comments.pdf [file pone.0354793.s001.pdf]

The manuscript presents a quantitative comparative analysis of the Mandarin verb-direction constructions **"V+ Xia," "V+ Xialai," and "V+ Xiaqu"** using collostructional analysis, semantic classification, and visualized distribution patterns. The topic is relevant to corpus-based construction grammar and contributes to the empirical study of Mandarin directional constructions. The study has a clear research focus, uses a large corpus, and attempts to move beyond intuition-based linguistic description by applying quantitative methods. Overall, the manuscript is valuable and suitable for publication after minor revision. The theoretical framework and quantitative approach are appropriate, but several sections would benefit from clearer methodological explanation, improved figure quality, and updated references.

Comments

1. The authors should provide more information on how the corpus examples were filtered and annotated. Since the study involves manual screening of cases where *Lai* and *Qu* do or do not function as directional verbs, it would be helpful to explain the annotation criteria more explicitly. If more than one annotator was involved, the authors should report inter-annotator agreement or briefly explain how disagreements were resolved.
2. The classification of semantic types is central to the manuscript. However, some categories, such as "completion/achievement," "state development," and "transition from dynamic to static," would benefit from clearer definitions and additional examples. This would make the classification more transparent and easier for readers to follow.
3. The statistical results are useful, but some interpretations could be more explicitly connected to the numerical findings. For example, when discussing productivity, collocational strength, and semantic prototypicality, the authors should briefly explain why the observed statistical patterns support the proposed linguistic conclusions.
4. The current figures are informative but should be improved for publication quality. The authors should increase figure resolution, enlarge axis labels and legends, improve color contrast, and ensure that Chinese labels are clearly readable.
5. The manuscript would benefit from the inclusion of more recent references, especially studies published in the last five years on usage-based construction grammar, collostructional analysis, quantitative corpus linguistics, Mandarin directional complements, and constructional networks. Adding recent literature would strengthen the theoretical background and show how the current work fits into ongoing research.

   The manuscript appears to have repeated or inconsistent table numbering in the section on distinctive collexeme analysis. The authors should carefully check the numbering of all tables and figures to ensure consistency, especially table 8-10.
6. The manuscript is generally understandable, but some sentences are long and dense. Minor language editing would improve clarity, especially in the theoretical framework and discussion sections. The authors should also ensure consistent formatting of terms such as *Xia*, *Xialai*, *Xiaqu*, "collocational strength," and "prototype meaning."

7. The manuscript states that corpus information is available from the BLCU Corpus Center. The authors should clarify whether the exact search queries, processed frequency tables, annotation data, or R scripts can be shared as supplementary materials. This would improve reproducibility.
