# Supplementary material for: Quantitative comparative study on the verb-direction constructions “V+ Xia (下)”, “V+ Xialai (下来)” and “V+ Xiaqu (下去)” in mandarin Chinese
Source: PLoS One. 2026 Jul 29;21(7):e0354793. doi: 10.1371/journal.pone.0354793 (PMC13419241; doi:10.1371/journal.pone.0354793)
Supplement: Attachment — Submitted filename: Response to Reviewers.docx [file pone.0354793.s002.docx]

**Response to Reviewers**

[PONE-D-25-52965]

Dear Dr. Arunkumar,

We would like to express our sincere gratitude for the chance to revise and resubmit our manuscript, “Quantitative comparative study on the verb-direction constructions ‘V+ Xia(下)’, ‘V+ Xialai(下来)’ and ‘V+ Xiaqu(下去)’ in Mandarin Chinese,” to *PLOS One*. We deeply appreciate the careful consideration and constructive feedback provided by you and the two reviewers. The detailed comments have been extremely helpful in refining and strengthening the paper.

We have incorporated most of the suggestions made by the reviewers. All modifications are clearly marked in the revised manuscript. Please find below our point-by-point responses (shown in blue) to each comment. Page numbers refer to the revised manuscript file with tracked changes.

**Reviewers’ Comments to the Author:**

**Reviewer 1**

This paper applies collexeme and distinctive collexeme analysis to compare the Mandarin verb-direction constructions “V+ Xia”, “V+ Xialai”, and “V+ Xiaqu”, using the BCC corpus. It identifies differences in collocational strength, prototype meanings, verb-type preferences, and semantic network structures via MDS. The study claims that “V+ Xia” has strongest entrenchment but lowest productivity, while “V+ Xialai” and “V+ Xiaqu” show broader productivity and distinct prototype semantics (“completion” vs. “continuation”). The paper is interesting and it can be published if the authors address the following concerns:

Author response: We sincerely thank the reviewer for the encouraging and positive assessment of our study, and for finding it interesting. We have carefully addressed all the concerns raised. Please find our detailed responses to each comment below.

1. How were non-directional homographs disambiguated automatically?

Author response: Thank you for this important point. We have clarified the procedure in the revised manuscript (see “Materials and methods”, Page 6). Non‑directional homographs were disambiguated through a three‑stage procedure. (1) Verb‑tag pre‑filtering: all queries required the slot‑filler to be verb‑tagged, automatically excluding noun‑ or adjective‑*Xia* sequences. (2) Filtering via the search results “Filter” function: we excluded the “*Zai*(在)...*Xia*” locative construction (queries “*Zai*nv*Xia*（在nv下）”, “*Zai*n*De*(的)v*Xia*（在n的v下）”) and the *Xia*‑as‑*Yixia*（一下） modal particle usage (queries “*Peihe*(配合)*Xia*”, “*Kaolv*（考虑）*Xia*”, etc.) directly in the filter bar. (3) Manual screening: the remaining few ambiguous cases were resolved through manual checking. The revised text now makes this procedure fully transparent.

2. How were split forms annotated computationally?

Author response: Thank you for this helpful reminder. In the original manuscript, the retrieval of potential and split forms was not clearly separated. We have now corrected this in the revised manuscript (see “Materials and methods”, Page 6), where the two types are described distinctly.

Split forms are constructions in which a locative or patient argument is inserted either within the directional verb or between the verb and the directional verb. Benefiting from the part-of-speech retrieval function of BCC corpus, these were retrieved using the queries “v*Xia*n*Lai*”(v下n来), “v*Xia*n*Qu*”(v下n去), “vn*XiaLai*”(vn下来), and “vn*XiaQu*”(vn下去). All retrieved results were then manually screened to exclude instances where Lai and Qu did not function as directional verbs.

3. Were passive constructions excluded?

Author response: We appreciate the reviewer’s careful consideration of whether passive constructions were covered by our retrieval method. Allow me to explain: passive forms were not excluded from the study.

Verb-direction constructions can freely occur in passive contexts in Mandarin Chinese. Two common forms are found in our data: (i) the overt passive, where the patient subject is followed by the passive marker *Bei* (被) and then the verb-direction construction, as in “杯子被碰下来了 (the cup was knocked down)”; and (ii) the unmarked passive, where the patient subject directly precedes the verb-direction construction, as in “这座城已攻下(this city has been conquered)”. Both types of passive forms would appear in our search results when retrieving verb-direction constructions. Therefore, passive forms are fully within the scope of this study.

4. How were frequency counts normalized across constructions of unequal base frequency?

Author response: We thank the reviewer for pointing out the imprecision in our discussion of collocational potential and productivity without normalization. In the revised manuscript, we have introduced the type‑token ratio (TTR) to address this issue (see “Results and discussion”, Page 12). TTR is calculated as the number of distinct verb types divided by the total number of tokens of that construction in the corpus. Two TTR values were computed for each construction: one using all collocatable verb types, and another using only verbs with a significant attraction relationship. The results confirm that “V+ *Xialai*” ranks highest (6.70‰ and 4.82‰), followed by “V+ *Xiaqu*” (6.20‰ and 4.31‰), and “V+ *Xia*” the lowest (0.73‰ and 0.46‰). Additionally, the collocational strength metric itself is inherently normalized, as Fisher’s exact test incorporates the total frequency of each construction in its calculation.

5. Why is productivity measured only via type count?

Author response: We recognize that raw type counts alone are an insufficient measure of productivity. As described in our response to Comment 4, we have now introduced the type‑token ratio (TTR) in the revised manuscript. The TTR based on verbs with a significant attraction relationship provides a normalized measure of productivity, and the results confirm the same pattern: “V+ *Xialai*” ranks highest (4.82‰), followed by “V+ *Xiaqu*” (4.31‰), and “V+ *Xia*” the lowest (0.46‰). We believe this provides a more robust basis for the productivity claims.

6. Was dispersion across texts calculated?

Author response: This is a helpful methodological point. We acknowledge that a systematic dispersion measure across texts was not calculated in the present study. Statistically counting the distribution of each specific construction across the various registers in a corpus as large as BCC would involve considerable workload and goes beyond the scope of this paper. We have noted this as a direction for future research in the revised manuscript (see “Conclusions”, Page 27).

Nevertheless, the validity of our findings is supported by two considerations. First, collostructional analysis via Fisher’s exact test requires a verb to occur in a construction with sufficient frequency relative to its overall corpus frequency to reach significance. Verbs whose occurrences are highly concentrated in a small number of texts are unlikely to pass this threshold, which provides an inherent safeguard against distributional bias. Second, “V+ *Xia*”, “V+ *Xialai*”, and “V+ *Xiaqu*” are among the most frequently used and stylistically neutral constructions in Mandarin Chinese, appearing naturally across a wide range of registers. The core findings are therefore unlikely to be artifacts of uneven text distribution.

7. Did you test whether collocational strength distributions differ significantly (e.g., via permutation tests)?

Author response: We think this is an excellent suggestion. We have now conducted permutation tests (10,000 iterations) in the revised manuscript (see “Results and discussion”, Page 13-14).

For the means, “V+ *Xia*” is significantly higher than both “V+ *Xialai*” and “V+ *Xiaqu*” (both one‑tailed p < 0.001), while “V+ *Xialai*” and “V+ *Xiaqu*” do not differ significantly (two‑tailed p = 0.790). For the standard deviations, “V+ *Xia*” is significantly more dispersed than “V+ *Xiaqu*” (one‑tailed p = 0.0016); the difference between “V+ *Xia*” and “V+ *Xialai*” shows a marginal trend but does not reach significance (one‑tailed p = 0.065). We have adjusted the relevant discussion accordingly.

8. Are the reported standard deviations statistically compared?

Author response: We thank the reviewer for this pertinent question. In the original manuscript, the reported standard deviations were not statistically compared. As noted in our response to Comment 7, the standard deviations of the three constructions have now been statistically compared via permutation tests (10,000 iterations). The results, reported in the revised manuscript (see “Results and discussion”, Page 11), show that “V+ Xia” is significantly more dispersed than “V+ Xiaqu” (p = 0.0016, one‑tailed), while the difference between “V+ Xia” and “V+ Xialai” shows a marginal trend but does not reach statistical significance (p = 0.065, one‑tailed).

9. Why was Fisher’s exact test preferred over log-likelihood ratio with large corpus size?

Author response: We appreciate this methodological question. Fisher's exact test was chosen because it is the standard statistical procedure in collostructional analysis as originally developed by Stefanowitsch and Gries (2003). Unlike the log‑likelihood ratio test, Fisher's exact test makes no distributional assumptions and computes exact p‑values via the hypergeometric distribution, which is particularly reliable for both low‑frequency and high‑frequency items. Given that our data include many low‑frequency verb‑construction pairs alongside high‑frequency ones, Fisher's exact test provides a consistent and conservative significance measure across the full frequency range. While the log‑likelihood ratio test is asymptotically equivalent to Fisher's exact test with large samples and could yield similar results, we followed the established practice in the collostructional literature to ensure methodological consistency and comparability with prior studies.

10. Did you adjust for multiple comparisons (hundreds of verbs tested)?

Author response: Thank you for pointing this out. The Benjamini‑Hochberg false discovery rate (FDR) correction was in fact applied during our computation in R (p_adj <- p.adjust(p_values, method = "BH")). However, we acknowledge that this step was not explicitly reported in the original manuscript, which was an oversight on our part. We have now added a clarification in the revised “Materials and methods” section (see Page 9). The numbers of significantly attracted verbs reported in the paper are all based on the FDR‑adjusted p‑values.

11. Why no Bayesian collostructional modeling?

Author response: We thank the reviewer for this insightful suggestion. Our use of the frequentist collostructional framework follows the standard procedure established by Stefanowitsch and Gries (2003), which has been widely applied in construction grammar studies. To further strengthen the validity of our findings, we have added a robustness check using mixed-effects models (see Section 4.4), the results of which show a very high consistency (Spearman’s ρ > 0.97) with the collocational strength rankings. This suggests our conclusions are highly stable regardless of the statistical framework employed. We fully agree that Bayesian models have already found meaningful applications in quantitative linguistic research, offering notable advantages such as providing credible intervals for parameter estimates and naturally addressing multiple comparisons through shrinkage. Exploring the application of Bayesian models to the verb-direction constructions studied here will be a valuable direction for our future research.

12. Did you consider conditional entropy instead of JSD?

Author response: We appreciate this thoughtful methodological question. We chose Jensen‑Shannon Divergence (JSD) for two main reasons. First, unlike Kullback-Leibler divergence, JSD is symmetric, which is an important property when comparing constructions without designating one as the reference point. Second, JSD is bounded, which yields more interpretable distances and facilitates the MDS visualization. Conditional entropy is an interesting alternative that could capture directional dependencies among the semantic types of the three constructions, and we see clear merit in exploring this in further analysis.

13. Why not use hierarchical clustering instead of visual MDS interpretation?

Author response: Thank you for raising this valuable point. MDS was chosen because our primary goal was to visualize the continuous gradient structure of the semantic networks and the relative distances among semantic types, which MDS represents effectively in a low‑dimensional space. Hierarchical clustering would be an excellent complementary method for identifying discrete groupings of semantic types, and we agree that adding it could enhance the robustness of the interpretation. We see this as a useful direction for further analysis of the semantic network structures of these constructions.

14. Are prototype distributions unimodal?

Author response: This is a helpful question. Based on visual inspection of the MDS plots, the prototype distributions of all three constructions appear unimodal, with scatter points tightly clustered around a single central region and no evidence of distinct subgroups. We have added a note to this effect in the revised manuscript (see “Results and discussion”, Page 24). This supports our interpretation that each construction has one coherent prototype meaning.

15. Could the high collocational strength of “V+ Xia” be an artifact of lower constructional frequency?

Author response: Thank you for this perceptive question. If we understand correctly, the concern is that the high collocational strength of “V+ *Xia*” may be driven by its highly concentrated distribution of verb types, namely, fewer types and larger token counts per type, rather than by genuinely tighter verb‑construction bonding.

We agree that this interpretative issue deserves careful examination. In the “V+ *Xia*” construction, a small number of verbs account for a large share of tokens, which naturally yields extremely low p‑values in Fisher’s exact test. However, two lines of evidence argue against treating the high collocational strength as a statistical artifact. First, collocational strength in this study is derived from Fisher’s exact test, which evaluates the deviation of observed frequency from expected frequency rather than raw frequency alone. A concentrated type distribution will only produce extremely low p‑values if the observed frequencies genuinely exceed what would be expected by chance, given the marginal frequencies. Second, the permutation tests reported earlier provide an independent statistical verification: the mean collocational strength of “V+ *Xia*” is significantly higher than those of “V+ *Xialai*” and “V+ *Xiaqu*”. This difference cannot be explained solely by the frequency profile of “V+ *Xia*”, as the permutation procedure controls for the distributional properties of each construction.

That said, we also note that from a usage‑based perspective, the concentrated distribution itself, which is independently confirmed by our TTR analysis, is consistent with the interpretation of high entrenchment. Rather than contradicting the collocational strength findings, the TTR results converge with them. Together, the permutation tests, the TTR analysis, and the nature of Fisher’s exact test provide converging evidence that the high collocational strength of “V+ *Xia*” reflects a genuine linguistic property of the construction, not merely a statistical consequence of its frequency profile.

16. Why no mixed-effects modeling controlling for verb lemma frequency?

Author response: We are grateful to the reviewer for raising this important methodological point. In the revised manuscript, we have added a new subsection “Robustness Check” (see “Results and discussion”, Page 24-27). In this subsection, we report a confirmatory analysis using mixed‑effects models to explicitly control for verb lemma frequency.

Specifically, for each construction (“V+ *Xia*”, “V+ *Xialai*”, “V+ *Xiaqu*”) we fitted an aggregated binomial generalized linear mixed model with the verb’s frequency in the construction and its total corpus frequency as the binomial dependent variable, the natural logarithm of the verb’s lemma frequency as the sole fixed effect, and random intercepts per verb. The random intercept for each verb represents its residual attraction after controlling for lemma frequency.

We then computed Spearman’s rank correlation coefficient ρ between the Fisher‑based collocational strength ranking and the random‑intercept ranking for each construction. The results (Table 11 in the revised manuscript) show that ρ = 0.978 for “V+ *Xia*”, 0.987 for “V+ *Xialai*”, and 0.988 for “V+ *Xiaqu*” (all p < 0.001). All ρ values are well above the 0.90 threshold for a “very strong correlation”, indicating that the ordering of verbs by attraction remains largely unchanged even after rigorous control for verb lemma frequency.

Table 12 presents 30 representative verbs with their random intercepts and original collocational strength values (full random‑intercept results for all attracted verbs are provided in Appendix 4). Within each construction, the ordering by random intercept is largely consistent with that by collocational strength. Verbs with positive random intercepts (e.g., *Ting*(停), *Zou*(走)) are core attractors that remain highly attractive after controlling for lemma frequency; verbs with negative random intercepts are located at the lower end of the ranking and often have very low collocational strength values, indicating that their apparent attraction is largely driven by low lemma frequency. Nevertheless, such negative‑intercept verbs remain significantly attracted according to the FDR‑corrected Fisher test and do not affect the main conclusions.

We believe this new analysis appropriately addresses the reviewer’s concern and confirms the robustness of our core findings. We greatly benefited from the reviewer’s suggestion to incorporate mixed‑effects modeling, which has significantly improved the methodological rigor of our study.

**Reviewer 2**

The manuscript presents a quantitative comparative analysis of the Mandarin verb-direction constructions “V+ Xia,” “V+ Xialai,” and “V+ Xiaqu” using collostructional analysis, semantic classification, and visualized distribution patterns. The topic is relevant to corpus-based construction grammar and contributes to the empirical study of Mandarin directional constructions. The study has a clear research focus, uses a large corpus, and attempts to move beyond intuition-based linguistic description by applying quantitative methods. Overall, the manuscript is valuable and suitable for publication after minor revision. The theoretical framework and quantitative approach are appropriate, but several sections would benefit from clearer methodological explanation, improved figure quality, and updated references.

Author response: We sincerely thank the reviewer for the thoughtful evaluation and for deeming our manuscript valuable and suitable for publication after minor revision. We are grateful for the specific guidance regarding the methodological explanation, figure quality, and references. We have carefully addressed all the points raised, and our detailed replies are presented below.

1. The authors should provide more information on how the corpus examples were filtered and annotated. Since the study involves manual screening of cases where Lai and Qu do or do not function as directional verbs, it would be helpful to explain the annotation criteria more explicitly. If more than one annotator was involved, the authors should report inter-annotator agreement or briefly explain how disagreements were resolved.

Author response: We appreciate this important suggestion. We have now added a detailed description of the annotation procedure in the revised manuscript (see “Materials and methods”, Page 6-7).

The manual screening and semantic type annotation were performed by two linguistically trained annotators. Before the formal annotation, both annotators received training on the criteria for identifying verb‑direction constructions, and a pilot annotation was conducted on 100 sample instances. Disagreements were discussed and resolved. Inter‑annotator agreement was satisfactory: Cohen's κ = 0.87 for construction identification and κ = 0.79 for semantic type classification.

We have also added two illustrative examples of common ambiguous cases. The first involves *Xia* functioning as a verbal measure word rather than a directional verb (e.g., “我的快递送到代收点了，你帮我取下它吧”, my express delivery has been delivered to the pickup point, could you please help me pick it up). The second involves structural ambiguity where “V+ *Xiaqu*” is not a “V+ *Xiaqu*” construction but rather a “V+ *Xia*” verb-direction construction followed by the purposive verb *Qu* in a serial verb construction (e.g., “把零件拆下去清洗”, disassemble the parts and clean them thoroughly). These examples are now included in the revised manuscript to clarify the annotation criteria.

2. The classification of semantic types is central to the manuscript. However, some categories, such as “completion/achievement,” “state development,” and “transition from dynamic to static,” would benefit from clearer definitions and additional examples. This would make the classification more transparent and easier for readers to follow.

Author response: Thank you for this constructive suggestion. We have substantially revised the manuscript by adding a detailed explanatory section immediately after Table 6, in which each of the seven semantic types is explicitly defined and illustrated with contextualized examples (see “Results and discussion”, Page 14-16). For semantic types shared across constructions (e.g., displacement from high to low, detachment, completion/achievement, state development), we provide comparative analyses specifying how subtle differences in semantic orientation and syntactic distribution lead to distinct constructional choices. For construction-specific types (contain, transition from dynamic to static, continuation of action), we clarify their unique semantic profiles and exemplify their usage. We believe these revisions make the classification more transparent and easier for readers to follow.

3. The statistical results are useful, but some interpretations could be more explicitly connected to the numerical findings. For example, when discussing productivity, collocational strength, and semantic prototypicality, the authors should briefly explain why the observed statistical patterns support the proposed linguistic conclusions.

Author response: We are grateful for this helpful suggestion. In the revised manuscript, we have added brief explanatory sentences at three points in the “Results and discussion” section to make the logical link between the numerical findings and the linguistic interpretations more explicit (see Page 12, 13, 17). Specifically, we now clarify that a higher TTR is the operational definition of greater productivity, as it indicates that a construction accommodates a wider range of verb types relative to its overall frequency; that higher collocational strength reflects a tighter and more habitual association between a construction and its verb slots, which in usage‑based theory corresponds to greater cognitive entrenchment; and that the semantic type accounting for the largest proportion of collocational strength is taken to be the prototype, as it represents the most frequently attested and cognitively most entrenched interpretation of the construction.

4. The current figures are informative but should be improved for publication quality. The authors should increase figure resolution, enlarge axis labels and legends, improve color contrast, and ensure that Chinese labels are clearly readable.

Author response: We sincerely thank the reviewer for the insightful suggestion. In response, all figures have been thoroughly revised to meet publication-quality standards (see “Results and discussion”, Page 9-11, 17, 19, 23). All figures are saved in SVG vector format, ensuring high resolution and clarity at any scale. Axis labels, tick labels, and legends have been substantially enlarged across all plots to improve readability to improve readability. for instance, axis titles were increased to 26 pt and tick labels to 22 pt. Color contrast has been enhanced by adopting high-saturation and perceptually uniform palettes: the collocational strength scatter plot now uses a custom red–orange–green–blue–purple gradient, the heatmap employs a warm white-to-deep-red gradient, and the remaining figures utilize carefully selected contrasting color schemes. For Chinese labels, we applied the bold SimHei font with significantly larger sizes (e.g., 10 pt in the scatter plot) and used repulsive text placement to avoid overlap, ensuring that all Chinese characters are clearly legible even when zoomed. These modifications have been applied consistently to all figures, including the collocational strength scatter plot, semantic heatmap, verb type bar chart, and semantic space map. We believe the revised figures now fully meet the publication requirements and have been updated in the revised manuscript.

5. The manuscript would benefit from the inclusion of more recent references, especially studies published in the last five years on usage-based construction grammar, collostructional analysis, quantitative corpus linguistics, Mandarin directional complements, and constructional networks. Adding recent literature would strengthen the theoretical background and show how the current work fits into ongoing research.

The manuscript appears to have repeated or inconsistent table numbering in the section on distinctive collexeme analysis. The authors should carefully check the numbering of all tables and figures to ensure consistency, especially table 8-10.

Author response: We appreciate the reviewer's beneficial‌ suggestions. In response, we have substantially updated the literature by incorporating several recent studies (published within the last five years) across the areas of usage-based construction grammar, collostructional analysis, quantitative corpus linguistics, and Mandarin directional complements (see “Introduction”, Page 3; “Theoretical framework”, Page 4-5; “Results and discussion”, Page 24-25). These additions have strengthened the theoretical background and better positioned our work within ongoing research. We have also carefully checked all tables and figures and corrected the inconsistent numbering in the distinctive collexeme analysis section (see “Results and discussion”, Page 20).

Specifically, in the Introduction, we have added Yang and Li (2025), a diachronic corpus-based study of the directional complement *Guolai* drawing on CCL data spanning fifteen historical periods, to illustrate the ongoing empirical shift in Chinese linguistics and to contrast with our synchronic, collostructional approach. In the Theoretical framework, we have incorporated Hilpert (2024), which surveys the future trajectories of Construction Grammar and reinforces our usage-based theoretical grounding. we have added Newman (2025) to validate the empirical utility of collostructional analysis across linguistic phenomena, and Liao, Gries and Wulff (2025) to demonstrate its successful application to Mandarin Chinese, thereby providing a direct methodological precedent for our study. In the newly added Robustness check, we have introduced a series of foundational and applied references on mixed-effects models—including Fisher (1918), Henderson (1950, 1953), Harville (1977), Laird and Ware (1982), Baayen (2008), and Gries (2015, 2021)—to ensure that our statistical validation is properly situated within the established methodological literature.

Regarding the table numbering issue, we have carefully reviewed all tables and found that Table 10 was erroneously labeled as “Table 9” in the original manuscript. This error has now been corrected, and all subsequent tables have been renumbered accordingly.

6. The manuscript is generally understandable, but some sentences are long and dense. Minor language editing would improve clarity, especially in the theoretical framework and discussion sections. The authors should also ensure consistent formatting of terms such as Xia, Xialai, Xiaqu, “collocational strength,” and “prototype meaning.”

Author response: We are grateful for this careful reading. In the revised manuscript, we have gone through the theoretical framework and discussion sections and shortened or restructured several long and dense sentences to improve readability. Two representative revisions are as follows: (1) In the theoretical framework, we reorganized the discussion of prototypes and frequency to make the logical connection more transparent (see Page 4). (2) In the discussion section, we streamlined the passage on the “completion/achievement” meanings of “V+ *Xia*” and “V+ *Xialai*” by disentangling the diachronic explanation from the synchronic comparison (see Page 21).

We have also checked the manuscript for terminological consistency. Specifically, the formatting of “V+ *Xia*”, “V+ *Xialai*”, and “V+ *Xiaqu*” has been standardized throughout; “collocation intensity” has been replaced by “collocational strength” in all figure and table headings; “prototypical meaning” has been unified to “prototype meaning” where it refers to the core semantic type; and “verb‑directional construction” has been replaced by “verb‑direction construction” for consistency with the paper’s title. These changes are reflected throughout the revised manuscript.

7. The manuscript states that corpus information is available from the BLCU Corpus Center. The authors should clarify whether the exact search queries, processed frequency tables, annotation data, or R scripts can be shared as supplementary materials. This would improve reproducibility.

Author response: We thank the reviewer for this suggestion regarding reproducibility. In response, we have compiled a comprehensive Supplementary Materials package and uploaded it to Figshare as a collection containing four items: (i) Appendix: collocational strength values and random-intercept results for all attracted verbs in the “V+ *Xia*”, “V+ *Xialai*”, and “V+ *Xiaqu*” constructions; (ii) Figures: all figures presented in the manuscript, provided in both PDF and SVG formats; (iii) Linguistic data: the complete datasets used for distinctive collexeme analysis, the verb-type bar charts, and the semantic-type heatmaps, including the frequency tables and calculated values beyond those reported in the Appendix; (iv) Code: all R code used for the statistical analyses reported in this study.

A private link has been generated for peer review purposes:

<https://figshare.com/s/0d86d164ef6c79bb7c27>

The collection will be made publicly available upon acceptance of the manuscript. We have also revised the Data Availability Statement in the manuscript to include this information.

Once again, we would like to express our sincere gratitude to the reviewer. Your valuable feedback has not only helped us strengthen the theoretical and methodological foundations of this work but has also greatly improved the overall quality of the manuscript. We have learned a great deal from this review process.
