# Supplementary material for: Quantitative comparative study on the verb-direction constructions “V+ Xia (下)”, “V+ Xialai (下来)” and “V+ Xiaqu (下去)” in mandarin Chinese
Source: PLoS One. 2026 Jul 29;21(7):e0354793. doi: 10.1371/journal.pone.0354793 (PMC13419241; doi:10.1371/journal.pone.0354793)
Supplement: Attachment — Submitted filename: Comment Sheet.docx [file pone.0354793.s003.docx]

**Review Comments to the Author**

I have reviewed the revised manuscript and the authors’ point-by-point response to the previous comments. The authors have made substantial and appropriate revisions, and the manuscript is now clearer and methodologically stronger.

The previous concerns regarding corpus filtering and annotation have been adequately addressed. The revised manuscript now explains the filtering procedure, the treatment of ambiguous cases, the role of the annotators, the pilot annotation process, and the inter-annotator agreement values. This improves the transparency and reliability of the corpus-based analysis.

The semantic classification has also been improved. The authors have added clearer definitions and contextualized examples for the major semantic categories, including categories such as completion/achievement, state development, and transition from dynamic to static. This makes the classification scheme easier to follow and better justified.

The statistical discussion is now stronger. The authors have added normalized productivity measures through type-token ratio, clarified the use of FDR-adjusted p-values, added permutation tests for comparing collocational strength distributions, and included a mixed-effects robustness check controlling for verb lemma frequency. These revisions provide a more rigorous basis for the linguistic conclusions.

The authors have also addressed the previous comments on figure quality, recent references, table numbering, terminology, language clarity, and reproducibility. The supplementary materials and code availability through Figshare are useful additions and improve the reproducibility of the study.

I have only one remaining minor editorial comment. The caption of Table 11 should be corrected. At present, it is labeled as “Data categories to be counted in collexeme analysis,” but the table actually reports Spearman’s rank correlation coefficients and p-values for the robustness check comparing Fisher-based collocational strength rankings with mixed-effects random-intercept rankings. The caption should be revised to accurately reflect the content of the table.

Subject to this minor correction, I consider the manuscript suitable for publication.
