# Supplementary material for: Quantitative comparative study on the verb-direction constructions “V+ Xia (下)”, “V+ Xialai (下来)” and “V+ Xiaqu (下去)” in mandarin Chinese
Source: PLoS One. 2026 Jul 29;21(7):e0354793. doi: 10.1371/journal.pone.0354793 (PMC13419241; doi:10.1371/journal.pone.0354793)
Supplement: Peer review history

# Peer review: Decision Letter 0

**DOI:** 10.1371/journal.pone.0354793.r001

**Authors:** ARUNKUMAR C

8 May 2026

PONE-D-25-52965

Quantitative comparative study on the verb-direction constructions “V+ Xia�下�”, “V+ Xialai�下来�” and “V+ Xiaqu�下去�” in mandarin Chinese

PLOS One

Dear Dr. Tan,

Thank you for submitting your manuscript to PLOS ONE. After careful consideration, we feel that it has merit but does not fully meet PLOS ONE’s publication criteria as it currently stands. Therefore, we invite you to submit a revised version of the manuscript that addresses the points raised during the review process.

Please submit your revised manuscript by Jun 22 2026 11:59PM. If you will need more time than this to complete your revisions, please reply to this message or contact the journal office at plosone@plos.org. When you're ready to submit your revision, log on to https://www.editorialmanager.com/pone/ and select the 'Submissions Needing Revision' folder to locate your manuscript file.

Please include the following items when submitting your revised manuscript:

- A letter that responds to each point raised by the academic editor and reviewer(s). You should upload this letter as a separate file labeled 'Response to Reviewers'.
- A marked-up copy of your manuscript that highlights changes made to the original version. You should upload this as a separate file labeled 'Revised Manuscript with Track Changes'.
- An unmarked version of your revised paper without tracked changes. You should upload this as a separate file labeled 'Manuscript'.

If you would like to make changes to your financial disclosure, please include your updated statement in your cover letter. Guidelines for resubmitting your figure files are available below the reviewer comments at the end of this letter.

If applicable, we recommend that you deposit your laboratory protocols in protocols.io to enhance the reproducibility of your results. Protocols.io assigns your protocol its own identifier (DOI) so that it can be cited independently in the future. For instructions see: https://journals.plos.org/plosone/s/submission-guidelines#loc-laboratory-protocols. Additionally, PLOS ONE offers an option for publishing peer-reviewed Lab Protocol articles, which describe protocols hosted on protocols.io. Read more information on sharing protocols at https://plos.org/protocols?utm_medium=editorial-email&utm_source=authorletters&utm_campaign=protocols.

As the corresponding author, your ORCID iD is verified in the submission system and will appear in the published article. PLOS supports the use of ORCID, and we encourage all coauthors to register for an ORCID iD and use it as well. Please encourage your coauthors to verify their ORCID iD within the submission system before final acceptance, as unverified ORCID iDs will not appear in the published article. *Only*  the individual author can complete the verification step; PLOS staff *cannot*  verify ORCID iDs on behalf of authors.

We look forward to receiving your revised manuscript.

Kind regards,

ARUNKUMAR C, Ph.D

Academic Editor

PLOS One

Journal Requirements:

When submitting your revision, we need you to address these additional requirements.

1. Please ensure that your manuscript meets PLOS ONE's style requirements, including those for file naming. The PLOS ONE style templates can be found at

https://journals.plos.org/plosone/s/file?id=wjVg/PLOSOne_formatting_sample_main_body.pdf and

https://journals.plos.org/plosone/s/file?id=ba62/PLOSOne_formatting_sample_title_authors_affiliations.pdf

2. Thank you for uploading your study's underlying data set. Unfortunately, the repository you have noted in your Data Availability statement does not qualify as an acceptable data repository according to PLOS's standards.

At this time, please upload the minimal data set necessary to replicate your study's findings to a stable, public repository (such as figshare or Dryad) and provide us with the relevant URLs, DOIs, or accession numbers that may be used to access these data. For a list of recommended repositories and additional information on PLOS standards for data deposition, please see https://journals.plos.org/plosone/s/recommended-repositories.

3. Please include a copy of Table 10 which you refer to in your text on page 23 (in PDF format).

4. If the reviewer comments include a recommendation to cite specific previously published works, please review and evaluate these publications to determine whether they are relevant and should be cited. There is no requirement to cite these works unless the editor has indicated otherwise.

[Note: HTML markup is below. Please do not edit.]

Reviewers' comments:

Reviewer's Responses to Questions

**Comments to the Author**

1. Is the manuscript technically sound, and do the data support the conclusions?

The manuscript must describe a technically sound piece of scientific research with data that supports the conclusions. Experiments must have been conducted rigorously, with appropriate controls, replication, and sample sizes. The conclusions must be drawn appropriately based on the data presented.

Reviewer #1: Yes

Reviewer #2: Yes

**********

2. Has the statistical analysis been performed appropriately and rigorously?

Reviewer #1: Yes

Reviewer #2: Yes

**********

3. Have the authors made all data underlying the findings in their manuscript fully available?

The PLOS Data policy requires authors to make all data underlying the findings described in their manuscript fully available without restriction, with rare exception (please refer to the Data Availability Statement in the manuscript PDF file). The data should be provided as part of the manuscript or its supporting information, or deposited to a public repository. For example, in addition to summary statistics, the data points behind means, medians and variance measures should be available. If there are restrictions on publicly sharing data—e.g. participant privacy or use of data from a third party—those must be specified.

Reviewer #1: No

Reviewer #2: Yes

**********

4. Is the manuscript presented in an intelligible fashion and written in standard English?

PLOS ONE does not copyedit accepted manuscripts, so the language in submitted articles must be clear, correct, and unambiguous. Any typographical or grammatical errors should be corrected at revision, so please note any specific errors here.

Reviewer #1: Yes

Reviewer #2: Yes

**********

5. Review Comments to the Author

Please use the space provided to explain your answers to the questions above. You may also include additional comments for the author, including concerns about dual publication, research ethics, or publication ethics. (Please upload your review as an attachment if it exceeds 20,000 characters)

Reviewer #1: This paper applies collexeme and distinctive collexeme analysis to compare the Mandarin verb-direction constructions “V+ Xia”, “V+ Xialai”, and “V+ Xiaqu”, using the BCC corpus. It identifies differences in collocational strength, prototype meanings, verb-type preferences, and semantic network structures via MDS. The study claims that “V+ Xia” has strongest entrenchment but lowest productivity, while “V+ Xialai” and “V+ Xiaqu” show broader productivity and distinct prototype semantics (“completion” vs. “continuation”). The paper is interesting and it can be published if the authors address the following concerns:

How were non-directional homographs disambiguated automatically?

How were split forms annotated computationally?

Were passive constructions excluded?

How were frequency counts normalized across constructions of unequal base frequency?

Why is productivity measured only via type count?

Was dispersion across texts calculated?

Did you test whether collocational strength distributions differ significantly (e.g., via permutation tests)?

Are the reported standard deviations statistically compared?

Why was Fisher’s exact test preferred over log-likelihood ratio with large corpus size?

Did you adjust for multiple comparisons (hundreds of verbs tested)?

Why no Bayesian collostructional modeling?

Did you consider conditional entropy instead of JSD?

Why not use hierarchical clustering instead of visual MDS interpretation?

Are prototype distributions unimodal?

Could the high collocational strength of “V+ Xia” be an artifact of lower constructional frequency?

Why no mixed-effects modeling controlling for verb lemma frequency?

Reviewer #2: The manuscript presents a quantitative comparative analysis of the Mandarin verb-direction constructions “V+ Xia,” “V+ Xialai,” and “V+ Xiaqu” using collostructional analysis, semantic classification, and visualized distribution patterns. The topic is relevant to corpus-based construction grammar and contributes to the empirical study of Mandarin directional constructions. The study has a clear research focus, uses a large corpus, and attempts to move beyond intuition-based linguistic description by applying quantitative methods. Overall, the manuscript is valuable and suitable for publication after minor revision. The theoretical framework and quantitative approach are appropriate, but several sections would benefit from clearer methodological explanation, improved figure quality, and updated references.

Comments

1. The authors should provide more information on how the corpus examples were filtered and annotated. Since the study involves manual screening of cases where Lai and Qu do or do not function as directional verbs, it would be helpful to explain the annotation criteria more explicitly. If more than one annotator was involved, the authors should report inter-annotator agreement or briefly explain how disagreements were resolved.

2. The classification of semantic types is central to the manuscript. However, some categories, such as “completion/achievement,” “state development,” and “transition from dynamic to static,” would benefit from clearer definitions and additional examples. This would make the classification more transparent and easier for readers to follow.

3. The statistical results are useful, but some interpretations could be more explicitly connected to the numerical findings. For example, when discussing productivity, collocational strength, and semantic prototypicality, the authors should briefly explain why the observed statistical patterns support the proposed linguistic conclusions.

4. The current figures are informative but should be improved for publication quality. The authors should increase figure resolution, enlarge axis labels and legends, improve color contrast, and ensure that Chinese labels are clearly readable.

5. The manuscript would benefit from the inclusion of more recent references, especially studies published in the last five years on usage-based construction grammar, collostructional analysis, quantitative corpus linguistics, Mandarin directional complements, and constructional networks. Adding recent literature would strengthen the theoretical background and show how the current work fits into ongoing research.

The manuscript appears to have repeated or inconsistent table numbering in the section on distinctive collexeme analysis. The authors should carefully check the numbering of all tables and figures to ensure consistency, especially table 8-10.

6. The manuscript is generally understandable, but some sentences are long and dense. Minor language editing would improve clarity, especially in the theoretical framework and discussion sections. The authors should also ensure consistent formatting of terms such as Xia, Xialai, Xiaqu, “collocational strength,” and “prototype meaning.”

7. The manuscript states that corpus information is available from the BLCU Corpus Center. The authors should clarify whether the exact search queries, processed frequency tables, annotation data, or R scripts can be shared as supplementary materials. This would improve reproducibility.

**********

6. PLOS authors have the option to publish the peer review history of their article (what does this mean?). If published, this will include your full peer review and any attached files.

If you choose “no”, your identity will remain anonymous but your review may still be made public.

**Do you want your identity to be public for this peer review?**  For information about this choice, including consent withdrawal, please see our Privacy Policy.

Reviewer #1: No

Reviewer #2: **Yes:** Aftab Anwar

**********

[NOTE: If reviewer comments were submitted as an attachment file, they will be attached to this email and accessible via the submission site. Please log into your account, locate the manuscript record, and check for the action link "View Attachments". If this link does not appear, there are no attachment files.]

To ensure your figures meet our technical requirements, please review our figure guidelines: https://journals.plos.org/plosone/s/figures

You may also use PLOS’s free figure tool, NAAS, to help you prepare publication quality figures: https://journals.plos.org/plosone/s/figures#loc-tools-for-figure-preparation.

NAAS will assess whether your figures meet our technical requirements by comparing each figure against our figure specifications.

Attachment Submitted filename: Comments.pdf

# Author comment: Author response to Decision Letter 1

**DOI:** 10.1371/journal.pone.0354793.r002

21 Jun 2026

Response to Reviewers

[PONE-D-25-52965]

Dear Dr. Arunkumar,

We would like to express our sincere gratitude for the chance to revise and resubmit our manuscript, “Quantitative comparative study on the verb-direction constructions ‘V+ Xia(下)’, ‘V+ Xialai(下来)’ and ‘V+ Xiaqu(下去)’ in Mandarin Chinese,” to PLOS One. We deeply appreciate the careful consideration and constructive feedback provided by you and the two reviewers. The detailed comments have been extremely helpful in refining and strengthening the paper.

We have incorporated most of the suggestions made by the reviewers. All modifications are clearly marked in the revised manuscript. Please find below our point-by-point responses (shown in blue) to each comment. Page numbers refer to the revised manuscript file with tracked changes.

Reviewers’ Comments to the Author:

Reviewer 1

This paper applies collexeme and distinctive collexeme analysis to compare the Mandarin verb-direction constructions “V+ Xia”, “V+ Xialai”, and “V+ Xiaqu”, using the BCC corpus. It identifies differences in collocational strength, prototype meanings, verb-type preferences, and semantic network structures via MDS. The study claims that “V+ Xia” has strongest entrenchment but lowest productivity, while “V+ Xialai” and “V+ Xiaqu” show broader productivity and distinct prototype semantics (“completion” vs. “continuation”). The paper is interesting and it can be published if the authors address the following concerns:

Author response: We sincerely thank the reviewer for the encouraging and positive assessment of our study, and for finding it interesting. We have carefully addressed all the concerns raised. Please find our detailed responses to each comment below.

1. How were non-directional homographs disambiguated automatically?

Author response: Thank you for this important point. We have clarified the procedure in the revised manuscript (see “Materials and methods”, Page 6). Non‑directional homographs were disambiguated through a three‑stage procedure. (1) Verb‑tag pre‑filtering: all queries required the slot‑filler to be verb‑tagged, automatically excluding noun‑ or adjective‑Xia sequences. (2) Filtering via the search results “Filter” function: we excluded the “Zai(在)...Xia” locative construction (queries “ZainvXia�在nv下�”, “ZainDe(的)vXia�在n的v下�”) and the Xia‑as‑Yixia�一下� modal particle usage (queries “Peihe(配合)Xia”, “Kaolv�考虑�Xia”, etc.) directly in the filter bar. (3) Manual screening: the remaining few ambiguous cases were resolved through manual checking. The revised text now makes this procedure fully transparent.

2. How were split forms annotated computationally?

Author response: Thank you for this helpful reminder. In the original manuscript, the retrieval of potential and split forms was not clearly separated. We have now corrected this in the revised manuscript (see “Materials and methods”, Page 6), where the two types are described distinctly.

Split forms are constructions in which a locative or patient argument is inserted either within the directional verb or between the verb and the directional verb. Benefiting from the part-of-speech retrieval function of BCC corpus, these were retrieved using the queries “vXianLai”(v下n来), “vXianQu”(v下n去), “vnXiaLai”(vn下来), and “vnXiaQu”(vn下去). All retrieved results were then manually screened to exclude instances where Lai and Qu did not function as directional verbs.

3. Were passive constructions excluded?

Author response: We appreciate the reviewer’s careful consideration of whether passive constructions were covered by our retrieval method. Allow me to explain: passive forms were not excluded from the study.

Verb-direction constructions can freely occur in passive contexts in Mandarin Chinese. Two common forms are found in our data: (i) the overt passive, where the patient subject is followed by the passive marker Bei (被) and then the verb-direction construction, as in “杯子被碰下来了 (the cup was knocked down)”; and (ii) the unmarked passive, where the patient subject directly precedes the verb-direction construction, as in “这座城已攻下(this city has been conquered)”. Both types of passive forms would appear in our search results when retrieving verb-direction constructions. Therefore, passive forms are fully within the scope of this study.

4. How were frequency counts normalized across constructions of unequal base frequency?

Author response: We thank the reviewer for pointing out the imprecision in our discussion of collocational potential and productivity without normalization. In the revised manuscript, we have introduced the type‑token ratio (TTR) to address this issue (see “Results and discussion”, Page 12). TTR is calculated as the number of distinct verb types divided by the total number of tokens of that construction in the corpus. Two TTR values were computed for each construction: one using all collocatable verb types, and another using only verbs with a significant attraction relationship. The results confirm that “V+ Xialai” ranks highest (6.70‰ and 4.82‰), followed by “V+ Xiaqu” (6.20‰ and 4.31‰), and “V+ Xia” the lowest (0.73‰ and 0.46‰). Additionally, the collocational strength metric itself is inherently normalized, as Fisher’s exact test incorporates the total frequency of each construction in its calculation.

5. Why is productivity measured only via type count?

Author response: We recognize that raw type counts alone are an insufficient measure of productivity. As described in our response to Comment 4, we have now introduced the type‑token ratio (TTR) in the revised manuscript. The TTR based on verbs with a significant attraction relationship provides a normalized measure of productivity, and the results confirm the same pattern: “V+ Xialai” ranks highest (4.82‰), followed by “V+ Xiaqu” (4.31‰), and “V+ Xia” the lowest (0.46‰). We believe this provides a more robust basis for the productivity claims.

6. Was dispersion across texts calculated?

Author response: This is a helpful methodological point. We acknowledge that a systematic dispersion measure across texts was not calculated in the present study. Statistically counting the distribution of each specific construction across the various registers in a corpus as large as BCC would involve considerable workload and goes beyond the scope of this paper. We have noted this as a direction for future research in the revised manuscript (see “Conclusions”, Page 27).

Nevertheless, the validity of our findings is supported by two considerations. First, collostructional analysis via Fisher’s exact test requires a verb to occur in a construction with sufficient frequency relative to its overall corpus frequency to reach significance. Verbs whose occurrences are highly concentrated in a small number of texts are unlikely to pass this threshold, which provides an inherent safeguard against distributional bias. Second, “V+ Xia”, “V+ Xialai”, and “V+ Xiaqu” are among the most frequently used and stylistically neutral constructions in Mandarin Chinese, appearing naturally across a wide range of registers. The core findings are therefore unlikely to be artifacts of uneven text distribution.

7. Did you test whether collocational strength distributions differ significantly (e.g., via permutation tests)?

Author response: We think this is an excellent suggestion. We have now conducted permutation tests (10,000 iterations) in the revised manuscript (see “Results and discussion”, Page 13-14).

For the means, “V+ Xia” is significantly higher than both “V+ Xialai” and “V+ Xiaqu” (both one‑tailed p < 0.001), while “V+ Xialai” and “V+ Xiaqu” do not differ significantly (two‑tailed p = 0.790). For the standard deviations, “V+ Xia” is significantly more dispersed than “V+ Xiaqu” (one‑tailed p = 0.0016); the difference between “V+ Xia” and “V+ Xialai” shows a marginal trend but does not reach significance (one‑tailed p = 0.065). We have adjusted the relevant discussion accordingly.

8. Are the reported standard deviations statistically compared?

Author response: We thank the reviewer for this pertinent question. In the original manuscript, the reported standard deviations were not statistically compared. As noted in our response to Comment 7, the standard deviations of the three constructions have now been statistically compared via permutation tests (10,000 iterations). The results, reported in the revised manuscript (see “Results and discussion”, Page 11), show that “V+ Xia” is significantly more dispersed than “V+ Xiaqu” (p = 0.0016, one‑tailed), while the difference between “V+ Xia” and “V+ Xialai” shows a marginal trend but does not reach statistical significance (p = 0.065, one‑tailed).

9. Why was Fisher’s exact test preferred over log-likelihood ratio with large corpus size?

Author response: We appreciate this methodological question. Fisher's exact test was chosen because it is the standard statistical procedure in collostructional analysis as originally developed by Stefanowitsch and Gries (2003). Unlike the log‑likelihood ratio test, Fisher's exact test makes no distributional assumptions and computes exact p‑values via the hypergeometric distribution, which is particularly reliable for both low‑frequency and high‑frequency items. Given that our data include many low‑frequency verb‑construction pairs alongside high‑frequency ones, Fisher's exact test provides a consistent and conservative significance measure across the full frequency range. While the log‑likelihood ratio test is asymptotically equivalent to Fisher's exact test with large samples and could yield similar results, we followed the established practice in the collostructional literature to ensure methodological consistency and comparability with prior studies.

10. Did you adjust for multiple comparisons (hundreds of verbs tested)?

Author response: Thank you for pointing this out. The Benjamini‑Hochberg false discovery rate (FDR) correction was in fact applied during our computation in R (p_adj <- p.adjust(p_values, method = "BH")). However, we acknowledge that this step was not explicitly reported in the original manuscript, which was an oversight on our part. We have now added a clarification in the revised “Materials and methods” section (see Page 9). The numbers of significantly attracted verbs reported in the paper are all based on the FDR‑adjusted p‑values.

11. Why no Bayesian collostructional modeling?

Author response: We thank the reviewer for this insightful suggestion. Our use of the frequentist collostructional framework follows the standard procedure established by Stefanowitsch and Gries (2003), which has been widely applied in construction grammar studies. To further strengthen the validity of our findings, we have added a robustness check using mixed-effects models (see Section 4.4), the results of which show a very high consistency (Spearman’s ρ > 0.97) with the collocational strength rankings. This suggests our conclusions are highly stable regardless of the statistical framework employed. We fully agree that Bayesian models have already found meaningful applications in quantitative linguistic research, offering notable advantages such as providing credible intervals for parameter estimates and naturally addressing multiple comparisons through shrinkage. Exploring the application of Bayesian models to the verb-direction constructions studied here will be a valuable direction for our future research.

12. Did you consider conditional entropy instead of JSD?

Author response: We appreciate this thoughtful methodological question. We chose Jensen‑Shannon Divergence (JSD) for two main reasons. First, unlike Kullback-Leibler divergence, JSD is symmetric, which is an important property when comparing constructions without designating one as the reference point. Second, JSD is bounded, which yields more interpretable distances and facilitates the MDS visualization. Conditional entropy is an interesting alternative that could capture directional dependencies among the semantic types of the three constructions, and we see clear merit in exploring this in further analysis.

13. Why not use hierarchical clustering instead of visual MDS interpretation?

Author response: Thank you for raising this valuable point. MDS was chosen because our primary goal was to visualize the continuous gradient structure of the semantic networks and the relative distances among semantic types, which MDS represents effectively in a low‑dimensional space. Hierarchical clustering would be an excellent complementary method for identifying discrete groupings of semantic types, and we agree that adding it could enhance the robustness of the interpretation. We see this as a useful direction for further analysis of the semantic network structures of these constructions.

14. Are prototype distributions unimodal?

Author response: This is a helpful question. Based on visual inspection of the MDS plots, the prototype distributions of all three constructions appear unimodal, with scatter points tightly clustered around a single central region and no evidence of distinct subgroups. We have added a note to this effect in the revised manuscript (see “Results and discussion”, Page 24). This supports our interpretation that each construction has one coherent prototype meaning.

15. Could the high collocational strength of “V+ Xia” be an artifact of lower constructional frequency?

Author response: Thank you for this perceptive question. If we understand correctly, the concern is that the high collocational strength of “V+ Xia” may be driven by its highly concentrated distribution of verb types, namely, fewer types and larger token counts per type, rather than by genuinely tighter verb‑construction bonding.

We agree that this interpretative issue deserves careful examination. In the “V+ Xia” construction, a small number of verbs account for a large share of tokens, which naturally yields extremely low p‑values in Fisher’s exact test. However, two lines of evidence argue against treating the high collocational strength as a statistical artifact. First, collocational strength in this study is derived from Fisher’s exact test, which evaluates the deviation of observed frequency from expected frequency rather than raw frequency alone. A concentrated type distribution will only produce extremely low p‑values if the observed frequencies genuinely exceed what would be expected by chance, given the marginal frequencies. Second, the permutation tests reported earlier provide an independent statistical verification: the mean collocational strength of “V+ Xia” is significantly higher than those of “V+ Xialai” and “V+ Xiaqu”. This difference cannot be explained solely by the frequency profile of “V+ Xia”, as the permutation procedure controls for the distributional properties of each construction.

That said, we also note that from a usage‑based perspective, the concentrated distribution itself, which is independently confirmed by our TTR analysis, is consistent with the interpretation of high entrenchment. Rather than contradicting the collocational strength findings, the TTR results converge with them. Together, the permutation tests, the TTR analysis, and the nature of Fisher’s exact test provide converging evidence that the high collocational strength of “V+ Xia” reflects a genuine linguistic property of the construction, not merely a statistical consequence of its frequency profile.

16. Why no mixed-effects modeling controlling for verb lemma frequency?

Author response: We are grateful to the reviewer for raising this important methodological point. In the revised manuscript, we have added a new subsection “Robustness Check” (see “Results and discussion”, Page 24-27). In this subsection, we report a confirmatory analysis using mixed‑effects models to explicitly control for verb lemma frequency.

Specifically, for each construction (“V+ Xia”, “V+ Xialai”, “V+ Xiaqu”) we fitted an aggregated binomial generalized linear mixed model with the verb’s frequency in the construction and its total corpus frequency as the binomial dependent variable, the natural logarithm of the verb’s lemma frequency as the sole fixed effect, and random intercepts per verb. The random intercept f

Attachment Submitted filename: Response to Reviewers.docx

# Peer review: Decision Letter 1

**DOI:** 10.1371/journal.pone.0354793.r003

**Authors:** ARUNKUMAR C

14 Jul 2026

Quantitative comparative study on the verb-direction constructions “V+ Xia�下�”, “V+ Xialai�下来�” and “V+ Xiaqu�下去�” in mandarin Chinese

PONE-D-25-52965R1

Dear Dr. %V+ Xia�下�%,

We’re pleased to inform you that your manuscript has been judged scientifically suitable for publication and will be formally accepted for publication once it meets all outstanding technical requirements.

Within one week, you’ll receive an e-mail detailing the required amendments. When these have been addressed, you’ll receive a formal acceptance letter and your manuscript will be scheduled for publication.

An invoice will be generated when your article is formally accepted. Please note, if your institution has a publishing partnership with PLOS and your article meets the relevant criteria, all or part of your publication costs will be covered. Please make sure your user information is up-to-date by logging into Editorial Manager at Editorial Manager® and clicking the ‘Update My Information' link at the top of the page. For questions related to billing, please contact billing support.

If your institution or institutions have a press office, please notify them about your upcoming paper to help maximize its impact. If they’ll be preparing press materials, please inform our press team as soon as possible -- no later than 48 hours after receiving the formal acceptance. Your manuscript will remain under strict press embargo until 2 pm Eastern Time on the date of publication. For more information, please contact onepress@plos.org.

Kind regards,

ARUNKUMAR C, Ph.D

Academic Editor

PLOS One

Additional Editor Comments (optional):

Reviewers' comments:

Reviewer's Responses to Questions

**Comments to the Author**

1. If the authors have adequately addressed your comments raised in a previous round of review and you feel that this manuscript is now acceptable for publication, you may indicate that here to bypass the “Comments to the Author” section, enter your conflict of interest statement in the “Confidential to Editor” section, and submit your "Accept" recommendation.

Reviewer #1: (No Response)

Reviewer #2: All comments have been addressed

**********

2. Is the manuscript technically sound, and do the data support the conclusions?

The manuscript must describe a technically sound piece of scientific research with data that supports the conclusions. Experiments must have been conducted rigorously, with appropriate controls, replication, and sample sizes. The conclusions must be drawn appropriately based on the data presented.

Reviewer #1: (No Response)

Reviewer #2: Yes

**********

3. Has the statistical analysis been performed appropriately and rigorously?

Reviewer #1: (No Response)

Reviewer #2: Yes

**********

4. Have the authors made all data underlying the findings in their manuscript fully available?

The PLOS Data policy requires authors to make all data underlying the findings described in their manuscript fully available without restriction, with rare exception (please refer to the Data Availability Statement in the manuscript PDF file). The data should be provided as part of the manuscript or its supporting information, or deposited to a public repository. For example, in addition to summary statistics, the data points behind means, medians and variance measures should be available. If there are restrictions on publicly sharing data—e.g. participant privacy or use of data from a third party—those must be specified.

Reviewer #1: (No Response)

Reviewer #2: Yes

**********

5. Is the manuscript presented in an intelligible fashion and written in standard English?

PLOS ONE does not copyedit accepted manuscripts, so the language in submitted articles must be clear, correct, and unambiguous. Any typographical or grammatical errors should be corrected at revision, so please note any specific errors here.

Reviewer #1: (No Response)

Reviewer #2: (No Response)

**********

6. Review Comments to the Author

Please use the space provided to explain your answers to the questions above. You may also include additional comments for the author, including concerns about dual publication, research ethics, or publication ethics. (Please upload your review as an attachment if it exceeds 20,000 characters)

Reviewer #1: (No Response)

Reviewer #2: I have reviewed the revised manuscript and the authors’ point-by-point response to the previous comments. The authors have made substantial and appropriate revisions, and the manuscript is now clearer and methodologically stronger.

The previous concerns regarding corpus filtering and annotation have been adequately addressed. The revised manuscript now explains the filtering procedure, the treatment of ambiguous cases, the role of the annotators, the pilot annotation process, and the inter-annotator agreement values. This improves the transparency and reliability of the corpus-based analysis.

The semantic classification has also been improved. The authors have added clearer definitions and contextualized examples for the major semantic categories, including categories such as completion/achievement, state development, and transition from dynamic to static. This makes the classification scheme easier to follow and better justified.

The statistical discussion is now stronger. The authors have added normalized productivity measures through type-token ratio, clarified the use of FDR-adjusted p-values, added permutation tests for comparing collocational strength distributions, and included a mixed-effects robustness check controlling for verb lemma frequency. These revisions provide a more rigorous basis for the linguistic conclusions.

The authors have also addressed the previous comments on figure quality, recent references, table numbering, terminology, language clarity, and reproducibility. The supplementary materials and code availability through Figshare are useful additions and improve the reproducibility of the study.

I have only one remaining minor editorial comment. The caption of Table 11 should be corrected. At present, it is labeled as “Data categories to be counted in collexeme analysis,” but the table actually reports Spearman’s rank correlation coefficients and p-values for the robustness check comparing Fisher-based collocational strength rankings with mixed-effects random-intercept rankings. The caption should be revised to accurately reflect the content of the table.

Subject to this minor correction, I consider the manuscript suitable for publication.

**********

7. PLOS authors have the option to publish the peer review history of their article (what does this mean?). If published, this will include your full peer review and any attached files.

If you choose “no”, your identity will remain anonymous but your review may still be made public.

**Do you want your identity to be public for this peer review?**  For information about this choice, including consent withdrawal, please see our Privacy Policy.

Reviewer #1: No

Reviewer #2: **Yes:** Aftab Anwar

**********

Attachment Submitted filename: Comment Sheet.docx

# Editor report: Acceptance letter

**DOI:** 10.1371/journal.pone.0354793.r004

**Authors:** ARUNKUMAR C

PONE-D-25-52965R1

PLOS One

Dear Dr. Tan,

I'm pleased to inform you that your manuscript has been deemed suitable for publication in PLOS One. Congratulations! Your manuscript is now being handed over to our production team.

At this stage, our production department will prepare your paper for publication. This includes ensuring the following:

* All references, tables, and figures are properly cited

* All relevant supporting information is included in the manuscript submission,

* There are no issues that prevent the paper from being properly typeset

You will receive further instructions from the production team, including instructions on how to review your proof when it is ready. Please keep in mind that we are working through a large volume of accepted articles, so please give us a few days to review your paper and let you know the next and final steps.

Lastly, if your institution or institutions have a press office, please let them know about your upcoming paper now to help maximize its impact. If they'll be preparing press materials, please inform our press team within the next 48 hours. Your manuscript will remain under strict press embargo until 2 pm Eastern Time on the date of publication. For more information, please contact onepress@plos.org.

You will receive an invoice from PLOS for your publication fee after your manuscript has reached the completed accept phase. If you receive an email requesting payment before acceptance or for any other service, this may be a phishing scheme. Learn how to identify phishing emails and protect your accounts at https://explore.plos.org/phishing.

If we can help with anything else, please email us at customercare@plos.org.

Thank you for submitting your work to PLOS ONE and supporting open access.

Kind regards,

PLOS ONE Editorial Office Staff

on behalf of

Dr. ARUNKUMAR C

Academic Editor

PLOS One
